# A Performance Comparison of Forcemyography and Electromyography in Muscle Force Estimation for Human–Machine Interfaces

**DOI:** 10.3390/s26206543

**Published:** 2026-10-15

**Authors:** Jessica Centracchio, Salvatore Parlato, Emilio Andreozzi, Eliana Cinotti, Paolo Bifulco, Gaetano Gargiulo, Daniele Esposito

**Affiliations:** 1 Department of Electrical Engineering and Information Technologies, University of Naples Federico II, Via Claudio, 21, I-80125 Naples, Italy; jessica.centracchio@unina.it (J.C.); salvatore.parlato@unina.it (S.P.);emilio.andreozzi@unina.it (E.A.); eliana.cinotti@unina.it (E.C.); paolo.bifulco@unina.it (P.B.); 2 School of Engineering, Design and Built Environment, Western Sydney University, Penrith, NSW 2747, Australia; g.gargiulo@westernsydney.edu.au; 3 Department of Information and Electrical Engineering and Applied Mathematics, University of Salerno, Via Giovanni Paolo II, 132, I-84084 Fisciano, Italy

**Keywords:** Forcemyography, FMG, Electromyography, EMG, muscle contraction, muscle force, Human–Machine Interface, HMI

## Abstract

**Highlights:**

**What are the main findings?**

- FMG achieved better mean force-estimation metrics than EMG-RMS, with significant advantages in selected comparisons, particularly during rapid isometric contractions.
- Subject-specific sigmoidal models estimated muscle force from FMG and EMG-RMS on held-out contractions.

**What are the implications of the main findings?**

- FMG avoids RMS-envelope extraction, offering a simpler preprocessing chain for force estimation.
- The results provide preliminary evidence for developing and validating FMG-based muscle-controlled HMIs, assistive exoskeletons, and rehabilitation devices.

**Abstract:**

The recording of muscle force can be used to control Human–Machine Interfaces (HMIs). A commonly used estimator of muscle force is the envelope of Electromyography (EMG) signals, which requires proper EMG processing. A potentially more convenient alternative is offered by Forcemyography (FMG), which records muscle mechanical activity and inherently provides a signal that is a good estimator of muscle force, without specific processing. This study aimed to compare the performances of EMG and FMG in muscle force estimation aimed at HMI control. Ten healthy subjects performed 15 gradual and 15 rapid isometric biceps brachii contractions at approximately 90° elbow flexion. Force measured at wrist by a load cell, and FMG and EMG signals were recorded simultaneously. Causal root-mean-square EMG envelopes (EMG-RMS) were computed offline using windows of 100, 250, 500, and 1000 ms. For each participant and contraction type, subject-specific sigmoidal regression models were fitted using normalized signals from the first 10 contractions and evaluated on the remaining five held-out contractions. Performance was assessed using the coefficient of determination (R^2^), root mean squared error (RMSE), and maximum absolute error (MaAE), with paired Wilcoxon tests and Bonferroni correction. Cross-correlation quantified temporal alignment with the reference force. FMG achieved higher mean R^2^ and lower mean RMSE and MaAE across all tested configurations. Statistical analyses on the errors revealed that FMG achieved performance comparable to that of EMG-RMS in gradual muscle contractions and significantly superior performance in rapid muscle contractions. These findings support FMG-based external force estimation with simpler pre-processing and provide preliminary evidence for future implementation and validation in muscle-controlled HMIs, assistive exoskeletons, and rehabilitation devices.

## 1. Introduction

Muscle force estimation is of interest in many research and application contexts. Evaluation of muscle performance in biomechanics research, monitoring of recovery progress during rehabilitation, prevention of work-related diseases in occupational medicine, diagnosis and therapy of neuromuscular disorders, assessment of sportive performance, and the implementation of force-proportional control in Human–Machine Interfaces (HMIs) are some representative examples [1,2,3,4].

A practical, noninvasive, indirect measure of muscle strength is obtained by means of surface Electromyography (sEMG), which provides information on muscle electrophysiological activity and is widely adopted in both research and applied settings [5,6,7]. The sEMG signal reflects depolarization of muscle fibers and can be recorded using skin-mounted electrodes. Its stochastic nature arises from the continuous variation in motor unit recruitment and firing rates during voluntary contractions [1,8,9]. To extract meaningful information, raw sEMG signals are typically processed through filtering stages aimed at reducing motion artifacts, power-line interference, and high-frequency noise [10]. Full-wave rectification followed by low-pass filtering is then applied to obtain the EMG envelope, which captures the average trend of muscle activation and is often used as an indirect estimator of muscle force [11].

However, the relationship between muscle force and the sEMG envelope is influenced by several physiological, anatomical, and technical factors, including subcutaneous fat thickness, electrode placement, muscle fiber orientation, crosstalk from adjacent muscles, and signal non-stationarity [1,12]. As a result, the EMG–force relationship is not universal and generally requires subject-specific and muscle-specific calibration [1,13,14,15,16,17]. These limitations become even more relevant in dynamic conditions, where joint kinematics and muscle geometry continuously change, further complicating the modeling of force generation [12]. For this reason, quantitative investigations of the EMG–force relationship are often conducted under isometric conditions, in which muscle tension is generated without significant changes in muscle length or joint movement [17]. In such conditions, externally measured forces or torques can be more reliably associated with muscle force output [8,17,18,19].

In real-time HMI applications, EMG-based force estimation relies on the EMG envelope, commonly obtained by calculating the EMG Root-Mean-Square value (EMG-RMS) over fixed time windows (0.25–1 s) [1,10,12], as recommended by the SENIAM project [11]. While these approaches improve signal stability, they introduce an inherent trade-off between accuracy and responsiveness. Longer smoothing windows reduce variability and noise but introduce non-negligible delays, whereas shorter windows improve temporal responsiveness at the cost of increased signal fluctuations [13]. This trade-off represents a critical limitation for real-time force estimation, where low latency and prompt tracking of muscle activation are essential.

Forcemyography (FMG) has recently gained attention as a complementary sensing modality for monitoring muscle contraction [20,21]. FMG is a non-invasive technique that uses force sensing elements to detect changes in muscles cross-section during contraction, providing information about mechanical phenomena of muscles in the low-frequency range (i.e., <10 Hz) [21,22]. The raw FMG signal was shown to be highly correlated with the EMG envelope acquired from the same muscle, and was effectively used for both muscle monitoring [23] and HMI applications [22,24,25,26,27,28,29,30,31].

Previous studies have compared FMG and EMG for various purposes such as motion classification, gesture recognition, and usability in real-time control scenarios. These studies generally reported improved robustness, repeatability, and user performance when using FMG-based approaches [32,33,34]. However, despite these promising findings, the role of FMG as a muscle force estimator and in direct comparison with EMG envelopes obtained through causal filtering, has not yet been fully explored.

The main contributions of this study are:

- A simplified biomechanical model relating the external force measured at the wrist to an equivalent biceps brachii force, providing a framework for interpreting the experimental measurements.
- A direct comparison of FMG and causal EMG-RMS for estimating the external force exerted at the wrist during gradual and rapid isometric contractions of the biceps brachii.
- The development and evaluation of subject-specific sigmoidal regression models for force estimation from FMG and EMG-RMS signals, using the force measured at the wrist as the reference and using separate sets of contractions for fitting and testing.
- Preliminary experimental evidence on FMG-based force estimation and its temporal alignment with the measured force, providing a basis for the future implementation and validation of FMG-based force-proportional Human–Machine Interfaces.

## 2. Materials and Methods

### 2.1. Biomechanical Model of the Biceps Brachii–Forearm System

**Figure 1:**
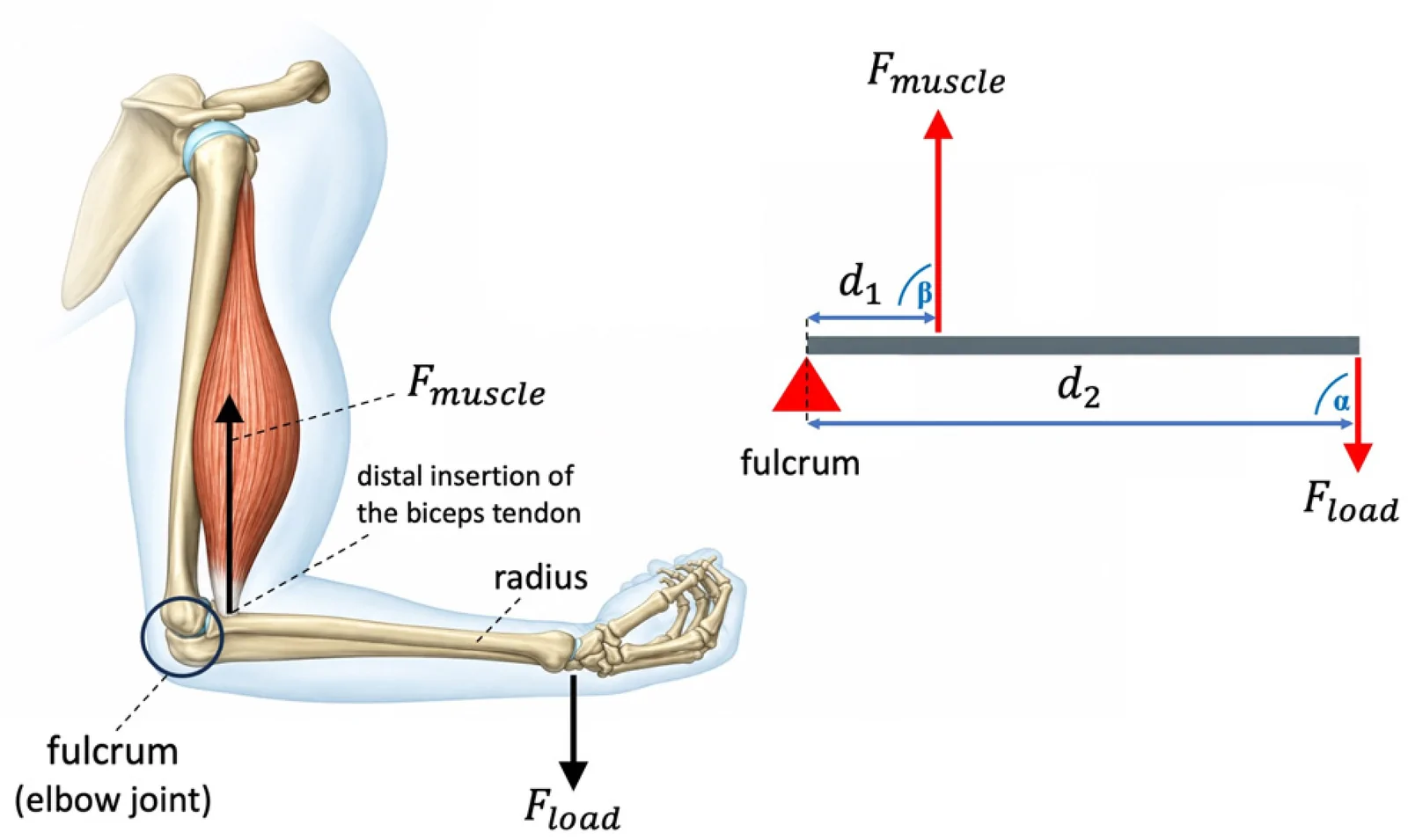
Anatomical illustration and simplified third-class lever model of the biceps brachii–forearm system. The muscle force acts at the radial tuberosity, between the elbow joint fulcrum and the load applied at the wrist. The distances “$d_{1}$” and “$d_{2}$” indicate the locations of muscle force and load application relative to the assumed fulcrum. Both forces are approximated as perpendicular to the radius (α = β = 90°). The illustration is not to scale.

The biceps brachii contributes to elbow flexion and forearm supination and also assists shoulder flexion [35]. During elbow flexion, it transmits force to the radius through its distal tendon, and the forearm functions as a third-class lever [36]. In this configuration, the muscle force is applied at the radial tuberosity, between the elbow joint, acting as the fulcrum, and the external load applied distally [35]. Figure 1 illustrates this configuration for the present study, in which the external force was applied at the wrist.

A simplified static model was adopted to describe the relationship between the external force measured at the wrist and an equivalent biceps brachii force. The model considers the opposing moments associated with these two forces, without explicitly accounting for the gravitational moments of the forearm and hand or the contributions of synergistic and antagonist muscles.

The articular margin of the radial head was adopted as an approximate anatomical reference for the elbow joint fulcrum. The distance ($d_{1}$) extends from this reference to the assumed longitudinal midpoint of the distal biceps’ tendon insertion. Athwal et al. [37] reported that the insertion begins, on average, 23 mm distal to the articular margin of the radial head and has a mean length of 21 mm. Assuming force application at the longitudinal midpoint of the insertion gives ($d_{1}=23+21/2=33.5$ mm). This value was adopted for all participants as a fixed geometric parameter of the simplified model. The distance “$d_{2}$”, approximately corresponding to forearm length, extends from the same anatomical reference to the point of force application at the wrist and was measured individually for each participant.

The angles α and β, defined between the radius and the load and muscle force directions, respectively, were both approximated as 90°. Under these assumptions, static equilibrium requires the opposing muscle and load moments [kgf·cm] to be equal in magnitude, as expressed in Equation (1):

(1) $$T_{muscle}=T_{load}\to F_{muscle}\times d_{1}=F_{load}\times d_{2}$$

Rearranging Equation (1) expresses the equivalent biceps brachii force ${(F}_{muscle})$ as a function of the measured external force ${(F}_{load})$ as expressed in Equation (2), where $k=\;\frac{d_{2}}{d_{1}}$ is a participant-specific scaling factor (values are reported in Table 1):

(2) $$F_{muscle}={\frac{d_{2}}{d_{1}}\;F}_{load}=kF_{load}$$

In the following experimental analyses, “reference force” denotes the external force measured at the wrist by the load cell ($F_{load}$), and its time course is referred to as the “reference force signal”. The term “estimated force” denotes the corresponding prediction obtained from FMG or EMG-RMS signals. For regression modelling and performance evaluation, ($F_{load}$) was retained as the experimentally measured reference. Accordingly, all force values and estimation errors reported in the figures and tables refer to ($F_{load}$), without applying the conversion in Equation (2).

### 2.2. Measurement Setup

The reference force was measured using a single-axis load cell conditioned by a custom circuit based on the INA125 instrumentation amplifier. The INA125 has an internal voltage reference that provided a stable supply voltage of 5 V to the Wheatstone bridge of the load cell. The conditioning circuit was powered with a 7.4 V battery pack and a differential amplification of 1542 V/V was set for the INA125 via a gain resistor of 39 Ω [38]. The load cell was calibrated with known weights to determine the weight/voltage transfer function. FMG was recorded using an Interlink FSR 402 Short Tail sensor, with a circular active sensing area 12.70 mm in diameter and a nominal thickness of 0.46 mm, as specified by the manufacturer [39]. A dome-shaped mechanical coupler with a base diameter of approximately 12 mm was positioned over the active sensing area. The sensor output was conditioned using a current mirror circuit powered by a 5 V supply and incorporating a 900 Ω gain resistor [29]. EMG measurements were performed via the BM623 biopotential amplifier (Biomedica Mangoni, Pisa, Italy), by setting a 5–500 Hz band-pass filter and a gain of 1000 V/V. A 50 Hz hardware notch filter was used to attenuate powerline noise. All signals (raw EMG, FMG and load cell signals) were simultaneously recorded at 1 kHz via a National Instruments NI-USB4431 DAQ board, with 24-bit resolution.

Ten healthy subjects (5 males and 5 females with an age of 24.6 ± 3.31 years) participated in the study after signing an informed consent form. The study was approved by the Ethical Committee of the University of Naples “Federico II” (ethical approval PG/2024/0026606). The characteristics of the participants are presented in Table 1.

**Figure 2:**
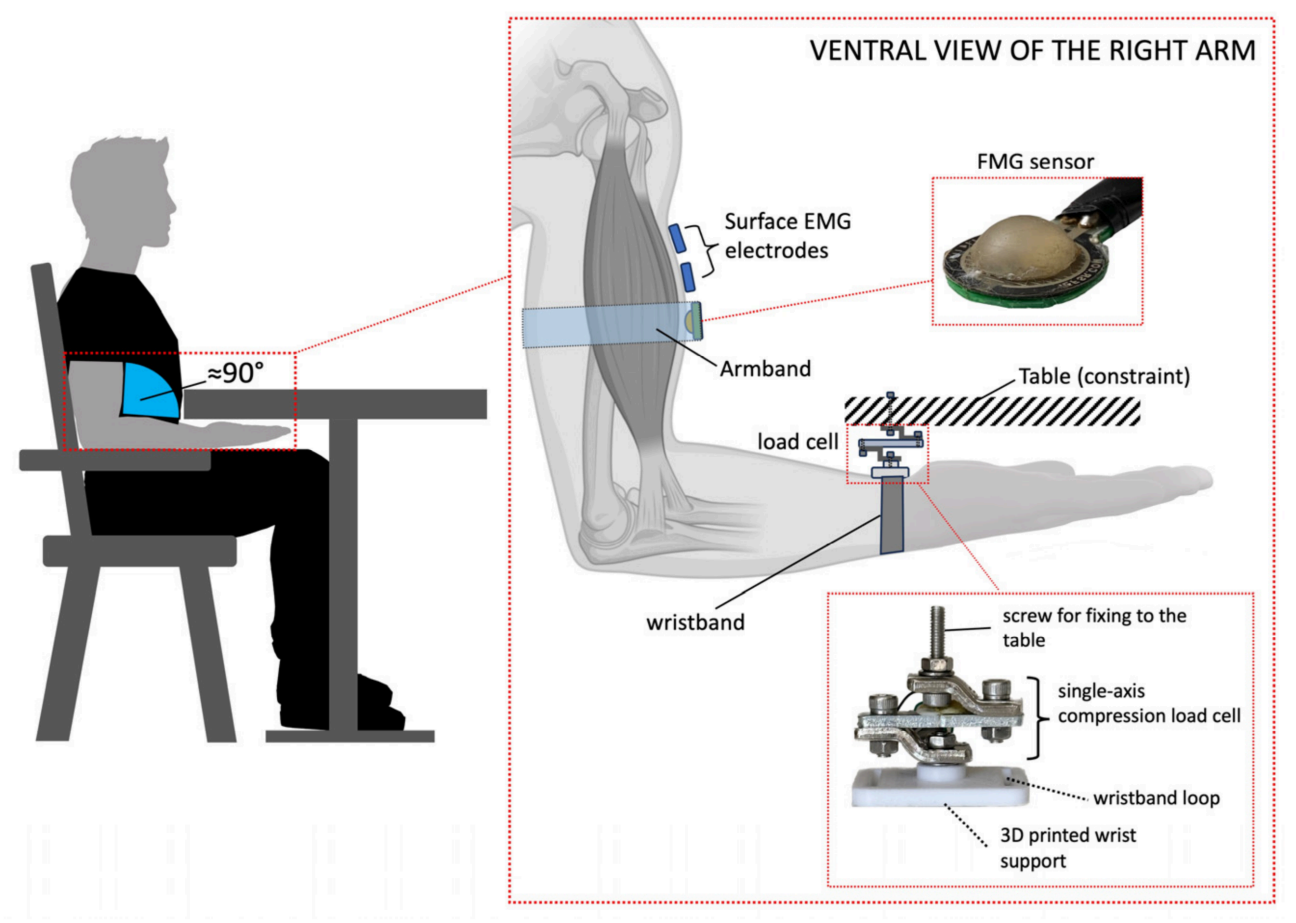
Experimental setup: the participant was seated with the elbow flexed at approximately 90° and instrumented with surface EMG electrodes, an FMG sensor, and a wristband connected to a load cell fixed to the table.

Each subject was comfortably seated in a chair with height adjustment and elbow support. A pair of surface EMG electrodes were applied on the biceps brachii following the recommendations of the SENIAM project [11]. The FMG sensor was positioned on the midline of the muscle belly, just below the pair of EMG electrodes. To this aim, the FMG was embedded in an elastic armband, which was wrapped around the muscle [29]. The armband was adjusted manually to maintain stable contact between the FMG sensor and the biceps brachii muscle without causing participant discomfort. No predefined contact-force target was imposed, and strap tension was not quantitatively standardized across participants. The single-axis load cell for reference force measurement was fixed to the lower surface of a table, which provided a reliable mechanical reference. Then, each participant wore a wrist band, which was attached to the load cell to prevent accidental detachments. Figure 2 illustrates the measurement setup.

### 2.3. Experimental Protocol

**Figure 3:**
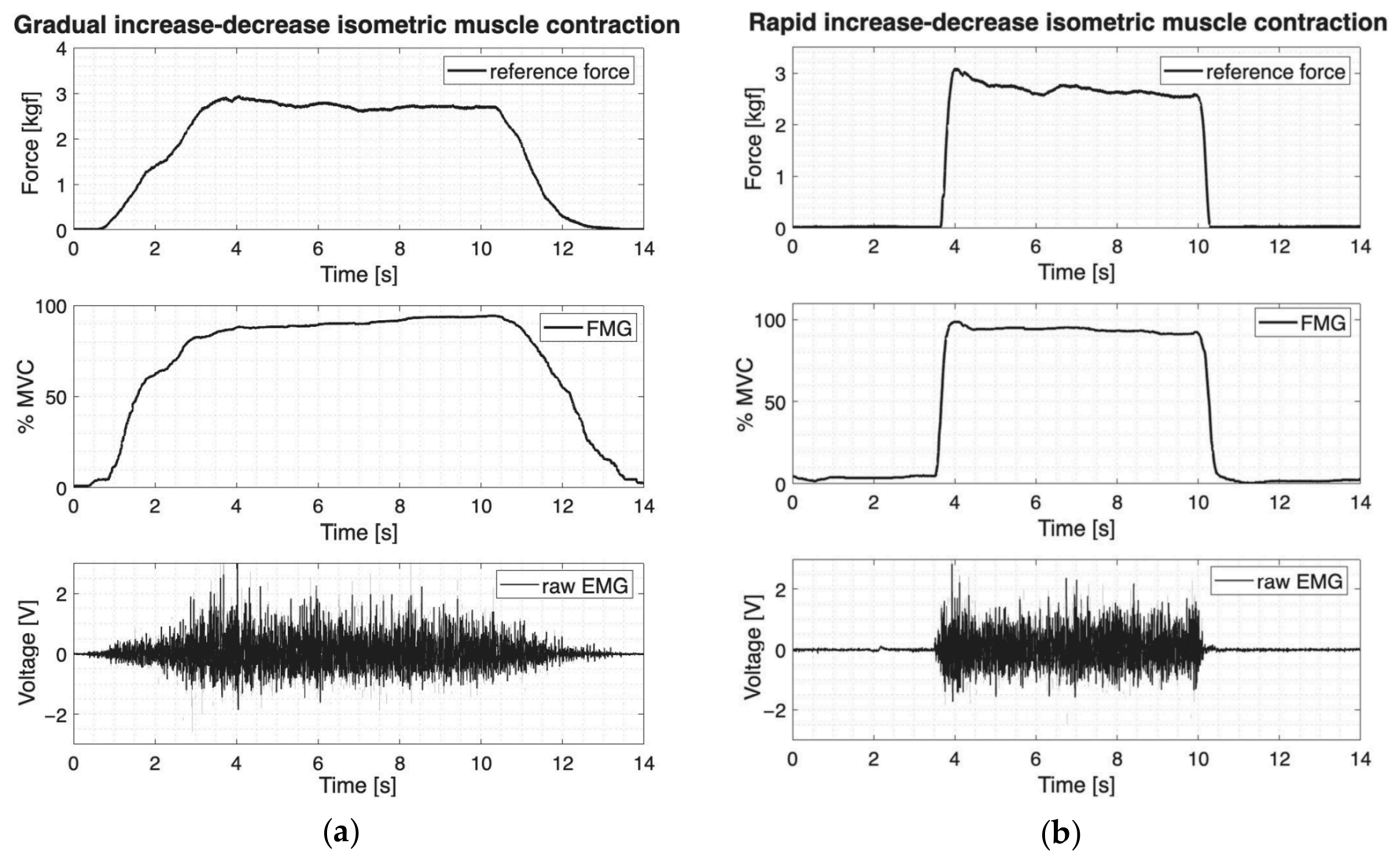
Representative simultaneous recordings of reference force, FMG, and raw EMG signals during isometric contractions of the biceps brachii in subject #3. (**a**) Gradual increase–decrease isometric contraction (Test A); (**b**) rapid increase–decrease isometric contraction (Test B). In each panel, the upper plot shows the external force measured at the wrist by the load cell (reference force, kgf), the middle plot shows the baseline-corrected and MVC-normalized FMG signal (%MVC), and the lower plot shows the raw EMG signal (V).

The subjects were asked to maintain the elbow flexed at about 90° while exerting force with the wrist against the load cell, so as to ensure that they performed isometric muscle contractions [40]. First, FMG and EMG values associated with Maximum Voluntary Contractions (MVC) were measured for each subject, according to the recommendations of the NeuroMuscular Research Center [9]. To this aim, the subjects were asked to exert their maximum force against the load cell for three times, letting them choose their own force rate to reach the maximal force. The MVC measures were then obtained by choosing the greatest value of the three attempts. After MVC measurements, two experimental tests were performed:

- (Test A) 15 gradual increase–decrease isometric muscle contractions characterized by a gradual increase in muscle contraction (lasting about 3 s); a sustained muscle contraction at an almost constant value (≈6 s); a gradual muscle relaxation, until the rest state was reached (≈3 s); and a total muscle relaxation (≈4 s) before the next contraction (see Figure 3a).
- (Test B) 15 rapid increase–decrease isometric muscle contractions characterized by a swift activation (lasting less than 0.5 s); a sustained muscle contraction at an almost constant value (≈6 s); a rapid relaxation until the rest state was reached (≈0.5 s); and a total muscle relaxation (≈4 s) before the next contraction (see Figure 3b).

Following MVC calibration, participants selected their own contraction intensity in both tests. No fixed percentage of MVC or common external-force target was prescribed. A graphical interface, designed with LabVIEW^®^ 2017 (National Instruments, Austin, TX, USA), displayed the recorded signals relative to MVC, while a video tutorial guided the temporal phases of each contraction. Test A was performed before Test B, with approximately five minutes of rest between the tests. No separate fatigue assessment was performed.

### 2.4. Signal Processing

Signal processing was performed offline on the recorded signals using MATLAB^®^ R2025b (The MathWorks, Natick, MA, USA). The FMG baseline offset associated with the static contact force between the sensor and the muscle was estimated from resting segments within the first 10 contractions used for model fitting. The same offset was subtracted from the MVC and test recordings. The corrected FMG signal was then normalized to the highest baseline-corrected amplitude across the three MVC trials. Baseline subtraction does not ensure identical mechanical coupling or sensor sensitivity across participants, and repeatability after sensor repositioning was not assessed.

The EMG signal was processed by subtracting its mean value calculated over the first 10 contractions. The same offset was applied to the corresponding MVC and test recordings. EMG envelopes were subsequently extracted using causal RMS windows of 100, 250, 500, and 1000 ms, with each output calculated from current and preceding samples. For each window length, the resulting EMG-RMS signal was normalized to the highest amplitude across the three MVC trials processed using that same window. All offsets and MVC references were determined without using the final five held-out test contractions and remained fixed during their evaluation.

**Figure 4:**
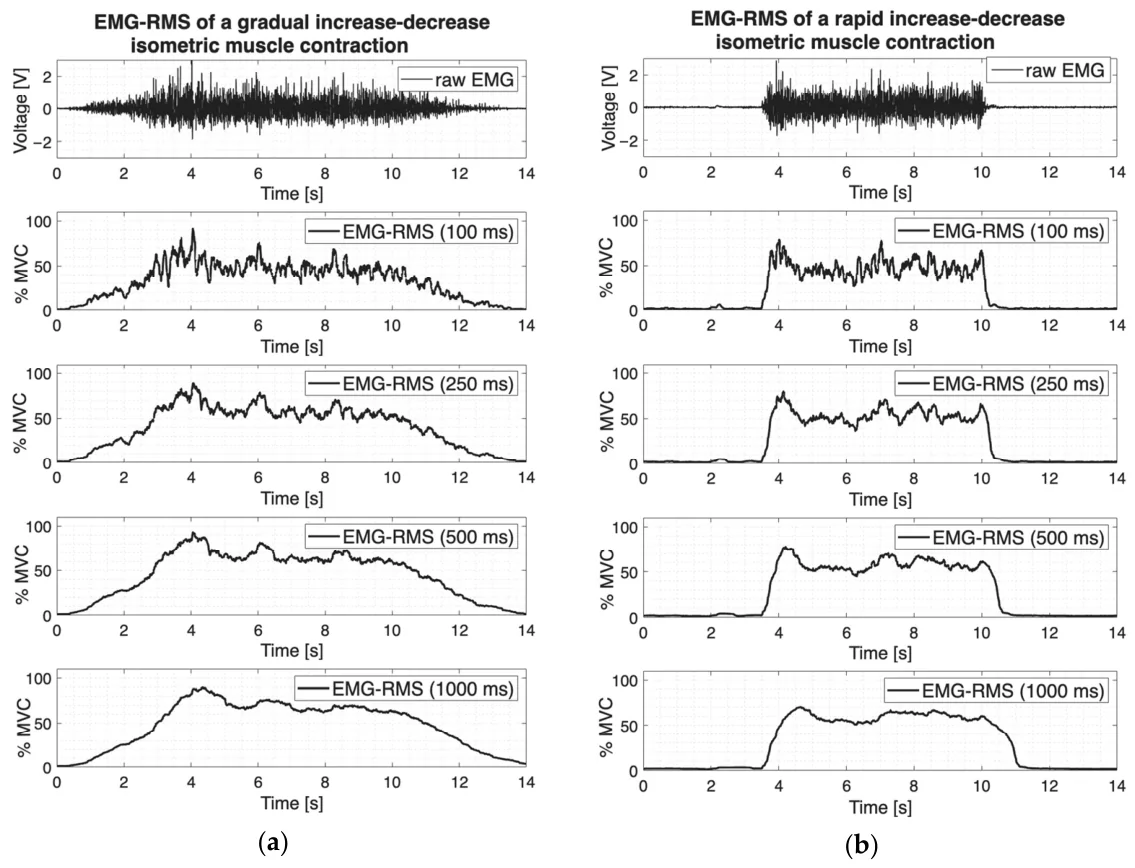
Examples of EMG-RMS signals (subject #3) computed on different time windows: (**a**) gradual increase–decrease isometric muscle contractions (Test A); (**b**) rapid increase–decrease isometric muscle contractions (Test B).

The four window lengths were selected to examine the influence of temporal smoothing on force-estimation performance during gradual and rapid contractions. As illustrated in Figure 4, longer windows produced smoother EMG-RMS envelopes but also more gradual rising and falling transitions.

The analysis evaluated force-estimation performance on recorded data using causal RMS-envelope extraction. Although this operation uses only current and preceding samples, causality alone does not establish the latency or timing performance of a complete real-time implementation. End-to-end acquisition-to-output latency, including acquisition, signal processing, and regression, was not measured.

### 2.5. Sigmoidal Regression Modelling and Evaluation

Both FMG and EMG-RMS signals were evaluated for estimating the force signal defined in Section 2.1. Previous studies have reported a nonlinear relationship between the EMG envelope and muscle force, particularly over a wide force range, which can be described by a sigmoidal function [15,41,42]. Accordingly, subject-specific sigmoidal regression models were fitted to relate the pre-processed FMG and EMG-RMS signals to the reference force signal. The sigmoidal model reported in Equation (3) was adopted:

(3) $$y\;=\;\frac{a}{1+e^{-b\left(x-c\right)}}$$

Here, (x) denotes the normalized FMG or EMG-RMS signal, and (y) denotes the corresponding estimated force, expressed in kgf. In this three-parameter logistic model, (a) defines the horizontal asymptote y=a, (b) controls the steepness and direction of the transition, and (c) denotes the value of x at the inflection point. At x=c, the output is y=a/2, corresponding to the midpoint between the two horizontal asymptotes, y=0 and y=a. The parameters (a), (b), and (c) were estimated using the MATLAB Curve Fitter app [43], together with their 95% confidence bounds.

For each participant and contraction type (test A and B), separate sigmoidal regression models were fitted for FMG and each EMG-RMS window length using the first 10 contractions. The model parameters were then held fixed, and the final 5 contractions were used exclusively for testing. The coefficient of determination (R^2^), the Root Mean Squared Error (RMSE), and the Maximum Absolute Error (MaAE) were computed on these 5 held-out contractions. This hold-out process assesses prediction for additional contractions of the same participant and type and does not assess generalization to new participants, different contraction types, or independent sessions.

### 2.6. Statistical Analysis

Statistical analyses were performed in MATLAB^®^ R2025b (The MathWorks, Natick, MA, USA) using participant-level R^2^, RMSE, and MaAE values computed on the held-out test contractions. FMG and EMG-RMS were compared using exact, two-sided paired Wilcoxon signed-rank tests, with observations paired by participant (*n* = 10 subjects). This nonparametric test does not require normally distributed data but assumes a symmetric distribution of the paired differences. Comparisons were performed for each metric and EMG-RMS window length (100, 250, 500, and 1000 ms) during gradual (Test A) and rapid (Test B) isometric muscle contractions.

For condition-specific analyses, Tests A and B were treated as separate families, each comprising 12 comparisons (three metrics × four RMS window lengths). Bonferroni correction was applied separately within each family by multiplying the unadjusted *p*-values by 12 and limiting the adjusted values to a maximum of 1. Statistical significance was defined as a Bonferroni-adjusted *p*-value < 0.05, equivalent to an unadjusted *p*-value < 0.05/12 (approximately 0.00417). This approach controls the family-wise error rate at 0.05 within each contraction condition.

To quantify the magnitude and uncertainty of the differences between methods, mean paired differences and their 95% confidence intervals (CIs) were calculated for each comparison. Within-participant differences were defined as FMG minus EMG-RMS for R^2^ and EMG-RMS minus FMG for RMSE and MaAE, so that positive values consistently indicated better FMG performance. Differences in R^2^ were dimensionless, whereas differences in RMSE and MaAE were expressed in kgf. Percentile bootstrap CIs were calculated using 50,000 resamples at the participant level. Each resample comprised 10 participants drawn with replacement, preserving the pairing between methods and using the same participant indices across all comparisons. The lower and upper CI bounds were the 2.5th and 97.5th percentiles of the bootstrap distribution of the mean paired difference. A fixed random seed ensured reproducibility. These CIs were pointwise and were not adjusted for multiple comparisons; statistical significance remained based on the Bonferroni-adjusted Wilcoxon *p*-values. Nonsignificant results were not interpreted as evidence of equivalence between methods.

### 2.7. Cross-Correlation Analysis of Force-Estimation Lags

Temporal alignment between the estimated force signals and the reference force measured by the load cell was assessed using cross-correlation in MATLAB^®^ R2025b (The MathWorks, Natick, MA, USA). The analysis was performed separately for gradual contractions (Test A) and rapid contractions (Test B), considering the FMG-based estimator and the EMG-RMS-based estimators with RMS windows of 100, 250, 500, and 1000 ms.

Individual contractions were manually segmented. For each contraction, a common time interval was applied to the reference signal and all five estimated force signals, including the rising and falling phases and adjacent resting portions. All five contractions belonging to held-out test dataset were analyzed per subject.

For each contraction and estimator, the lag corresponding to the maximum cross-correlation value was extracted and converted from samples to milliseconds using the sampling frequency. Positive lags indicate that the estimated force is delayed relative to the reference force, whereas negative lags indicate an advance.

For each subject, test, and estimator, the mean, standard deviation, and median of the contraction-level lags were calculated. Group results were subsequently summarized as the unweighted mean and standard deviation of the 10 subject-specific mean lags.

## 3. Results

### 3.1. Results of Test A: Gradual Increase–Decrease Isometric Muscle Contractions

#### 3.1.1. Force Signal vs. FMG Signal

Across all ten subjects, the subject-specific sigmoidal regression models relating the FMG signal to the force signal during Test A achieved a mean coefficient of determination (R^2^) of 0.922 ± 0.047, a Root Mean Squared Error (RMSE) of 0.422 ± 0.158 kgf, and a Maximum Absolute Error (MaAE) of 1.360 ± 0.351 kgf. The subject-specific prediction performance metrics are reported in Table S1 of the Supplementary Materials.

**Figure 5:**
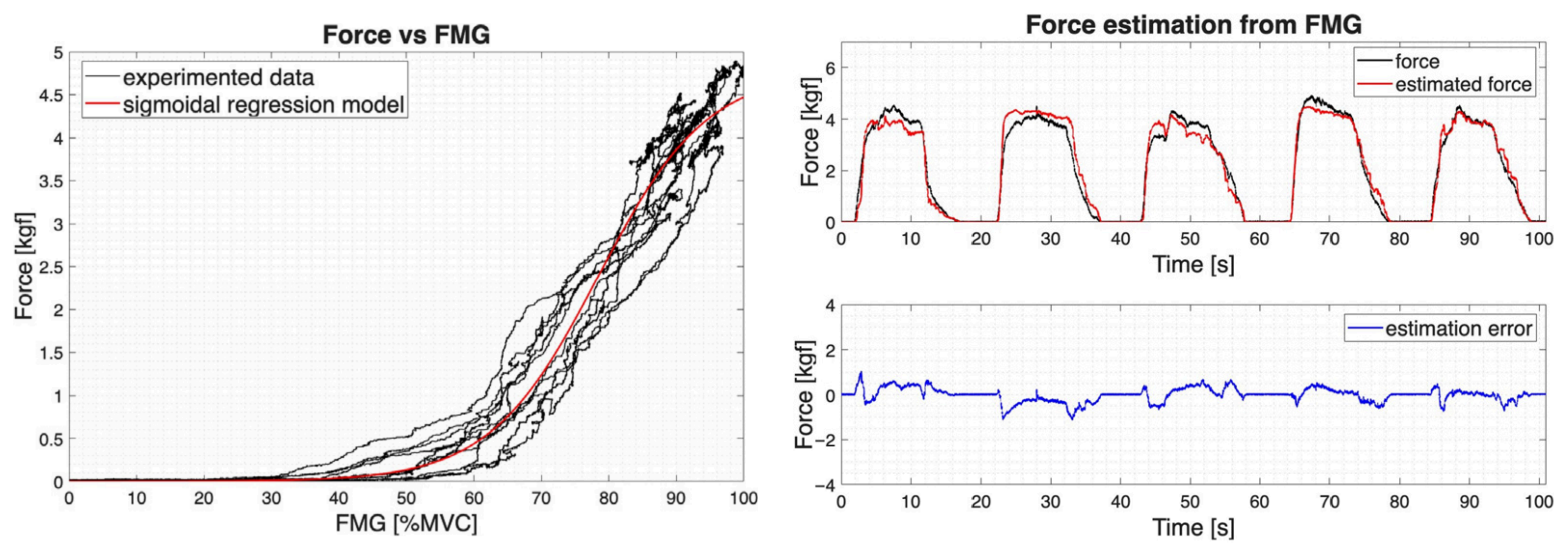
Subject-specific FMG-based force estimation for subject #3 during the five held-out test contractions of Test A (gradual increase–decrease isometric contractions). Left: relationship between normalized FMG and reference force for the five test contractions (black), together with the sigmoidal regression curve fitted using the first 10 contractions (red). Upper right: comparison between the reference force (“force”, black) and the model prediction (“estimated force”, red). Bottom right: estimation error over time, calculated as reference force minus FMG-based estimated force.

Figure 5 illustrates the performance of the subject-specific FMG regression model for subject #3 during Test A. The left panel shows the relationship between the normalized FMG signal and the force signal, together with the fitted sigmoidal regression model. The right panels show the corresponding force estimation results: the upper panel compares the force signal with the FMG-based estimated force, while the bottom panel reports the estimation error over time, computed as the difference between the force signal and the FMG-based estimated force.

#### 3.1.2. Force Signal vs. EMG-RMS Signal

Across all ten subjects, the prediction performance metrics obtained from the subject-specific sigmoidal regression models relating the force signal to the EMG-RMS signal during Test A are summarized in Table 2 for each RMS time window considered. The best results were achieved with a time window of 500 ms, which resulted in the highest coefficient of determination (R^2^ = 0.893 ± 0.049) and the lowest estimation errors (RMSE = 0.489 ± 0.128 kgf and MaAE = 1.844 ± 0.397 kgf). The detailed results of the EMG-RMS performance during Test A for each subject are reported in Table S2 in the Supplementary Materials.

**Figure 6:**
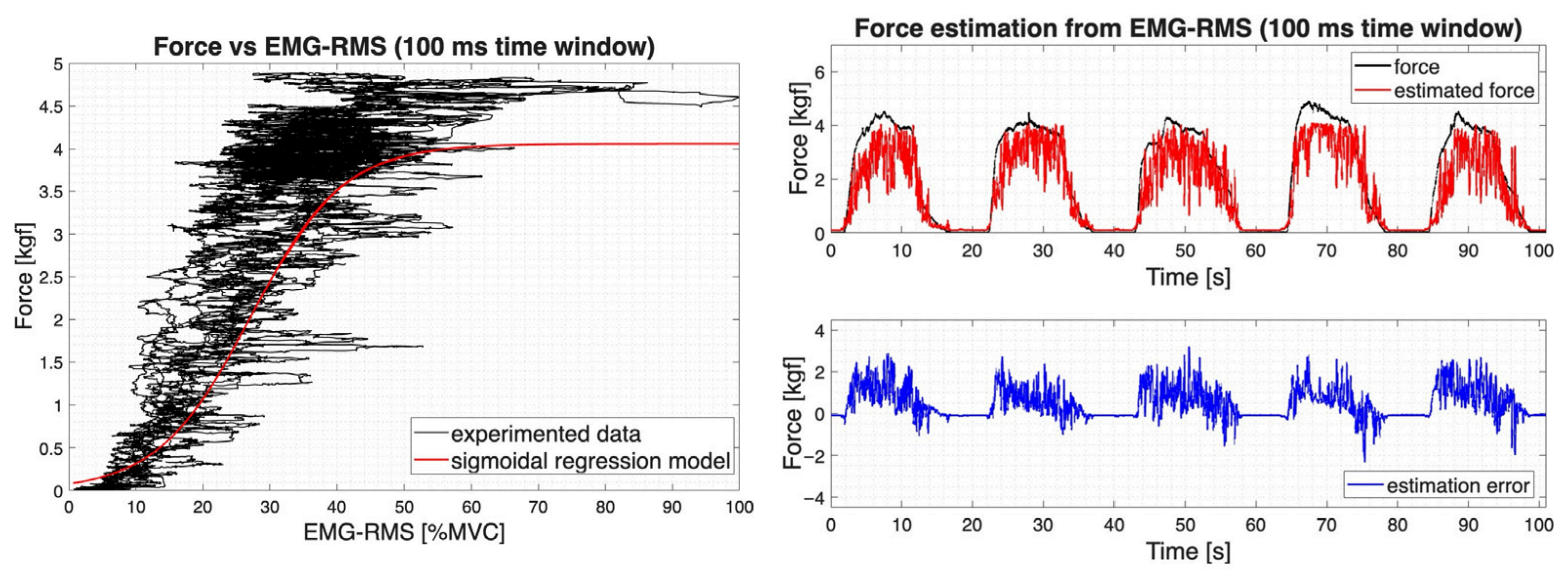
Subject-specific EMG-RMS-based force estimation, using a 100 ms RMS time window for subject #3 during the five held-out test contractions of Test A (gradual increase–decrease isometric contractions). Left: relationship between normalized EMG-RMS and reference force for the five test contractions (black), together with the sigmoidal regression curve fitted using the first 10 contractions (red). Upper right: comparison between the measured force (“force”, black) and the model prediction (“estimated force”, red). Bottom right: estimation error over time, calculated as reference force minus EMG-RMS-based estimated force.

**Figure 7:**
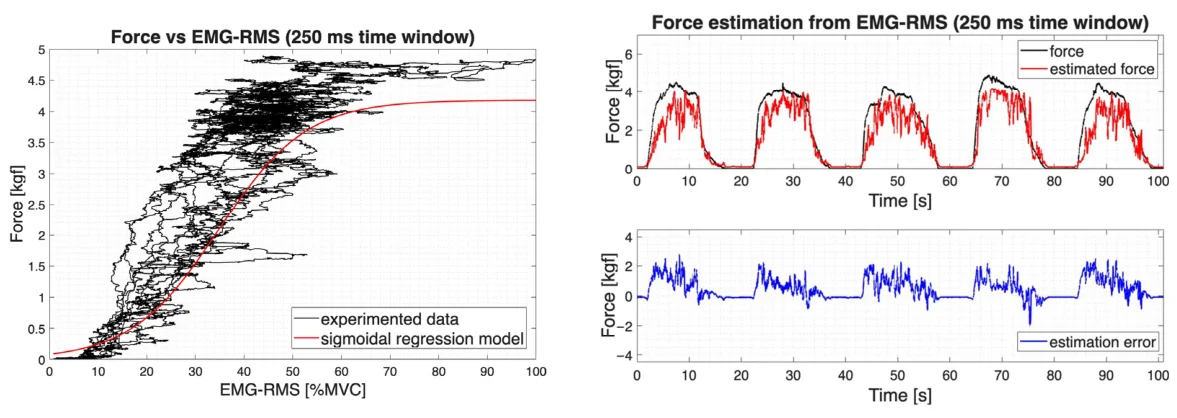
Subject-specific EMG-RMS-based force estimation, using a 250 ms RMS time window for subject #3 during the five held-out test contractions of Test A (gradual increase–decrease isometric contractions). Left: relationship between normalized EMG-RMS and measured force for the five test contractions (black), together with the sigmoidal regression curve fitted using the first 10 contractions (red). Upper right: comparison between the reference force (“force”, black) and the model prediction (“estimated force”, red). Bottom right: estimation error over time, calculated as reference force minus EMG-RMS-based estimated force.

**Figure 8:**
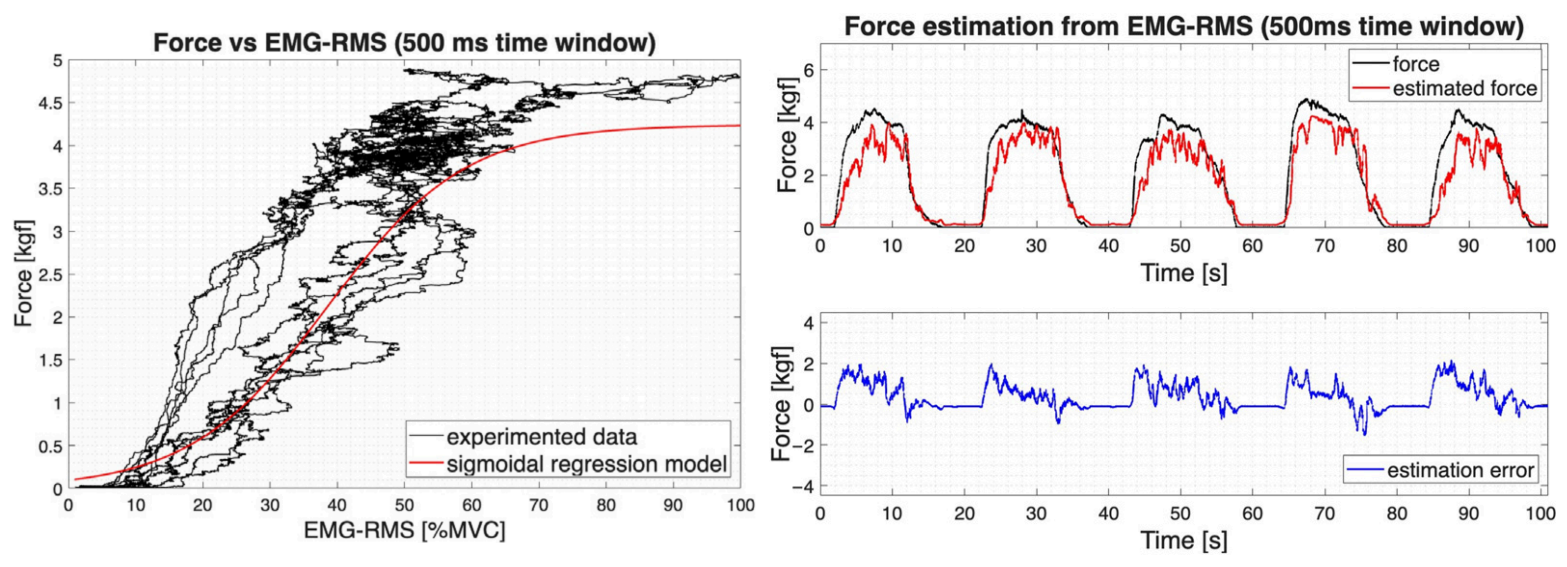
Subject-specific EMG-RMS-based force estimation, using a 500 ms RMS time window for subject #3 during the five held-out test contractions of Test A (gradual increase–decrease isometric contractions). Left: relationship between normalized EMG-RMS and measured force for the five test contractions (black), together with the sigmoidal regression curve fitted using the first 10 contractions (red). Upper right: comparison between the reference force (“force”, black) and the model prediction (“estimated force”, red). Bottom right: estimation error over time, calculated as reference force minus EMG-RMS-based estimated force.

**Figure 9:**
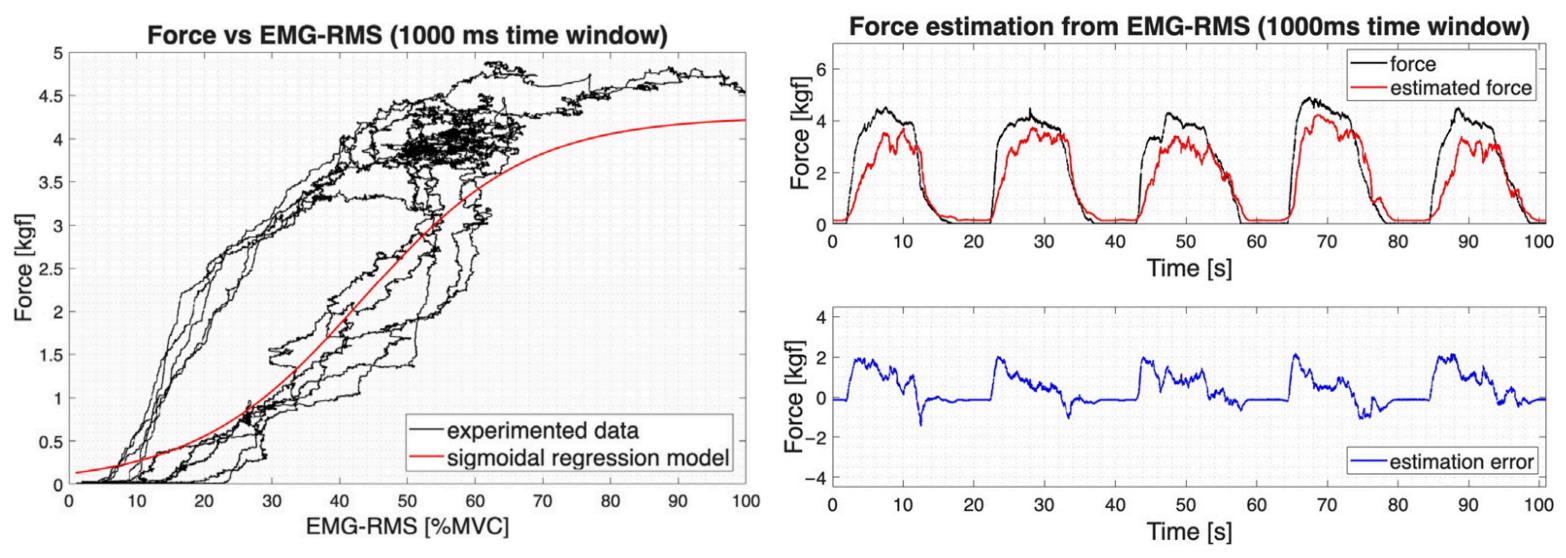
Subject-specific EMG-RMS-based force estimation, using a 1000 ms RMS time window for subject #3 during the five held-out test contractions of Test A (gradual increase–decrease isometric contractions). Left: relationship between normalized EMG-RMS and measured force for the five test contractions (black), together with the sigmoidal regression curve fitted using the first 10 contractions (red). Upper right: comparison between the reference force (“force”, black) and the model prediction (“estimated force”, red). Bottom right: estimation error over time, calculated as reference force minus EMG-RMS-based estimated force.

Figure 6, Figure 7, Figure 8 and Figure 9 illustrate the performance of the subject-specific EMG-RMS regression models for subject #3 during Test A. In each figure, the left panel shows the relationship between the normalized EMG-RMS signal, computed using time windows ranging from 100 ms to 1000 ms, and the force signal, together with the fitted sigmoidal regression model. The right panels show the corresponding force estimation results: the upper panel compares the force signal with the EMG-RMS-based estimated force, while the bottom panel reports the estimation error over time, computed as the difference between the force signal and the EMG-RMS-based estimated force.

### 3.2. Results of Test B—Rapid Increase–Decrease Isometric Muscle Contractions

#### 3.2.1. Force Signal vs. FMG Signal

Across all ten subjects, the subject-specific sigmoidal regression models relating the FMG signal to the force signal during Test B achieved a mean coefficient of determination (R^2^) of 0.943 ± 0.016, a Root Mean Squared Error (RMSE) of 0.382 ± 0.170 kgf, and a Maximum Absolute Error (MaAE) of 2.029 ± 1.159 kgf. The subject-specific prediction performance metrics are reported in Table S3 of the Supplementary Materials.

**Figure 10:**
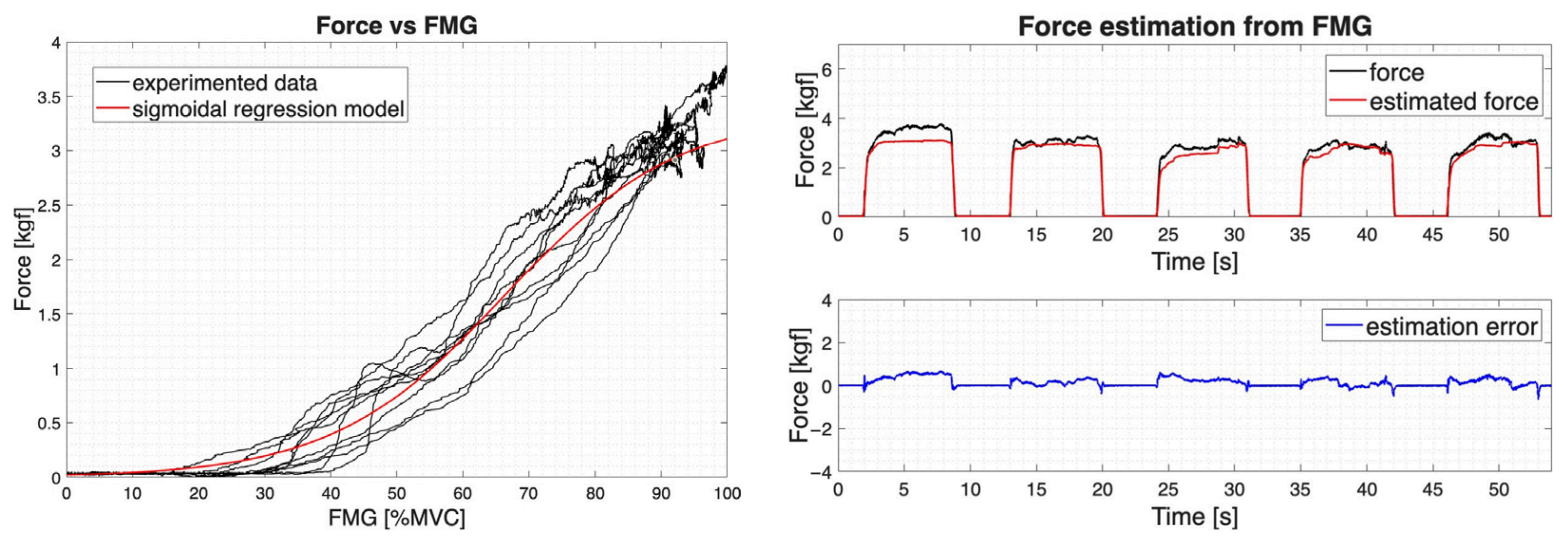
Subject-specific FMG-based force estimation for subject #5 during the five held-out test contractions of Test B (rapid increase–decrease isometric contractions). Left: relationship between normalized FMG and measured force for the five test contractions (black), together with the sigmoidal regression curve fitted using the first 10 contractions (red). Upper right: comparison between the reference force (“force”, black) and the model prediction (“estimated force”, red). Bottom right: estimation error over time, calculated as reference force minus FMG-based estimated force.

Figure 10 illustrates the performance of the subject-specific FMG regression model for subject #5 during Test B. The left panel shows the relationship between the normalized FMG signal and the force signal, together with the fitted sigmoidal regression model. The right panels show the corresponding force estimation results: the upper panel compares the force signal with the FMG-based estimated force, while the bottom panel reports the estimation error over time, computed as the difference between the force signal and the FMG-based estimated force.

#### 3.2.2. Force Signal vs. EMG-RMS Signal

Across all ten subjects, the prediction performance metrics obtained from the subject-specific sigmoidal regression models relating the force signal to the EMG-RMS signal during test B are summarized in Table 3 for each RMS time window considered. The best results were achieved with the time window of 250 ms, which resulted in the highest coefficient of determination (R^2^ = 0.918 ± 0.024) and the lowest estimation errors (RMSE = 0.440 ± 0.147 kgf and MaAE = 2.778 ± 1.189 kgf). The detailed results of the EMG-RMS performance during Test B for each subject are reported in Table S4 in the Supplementary Materials.

**Figure 11:**
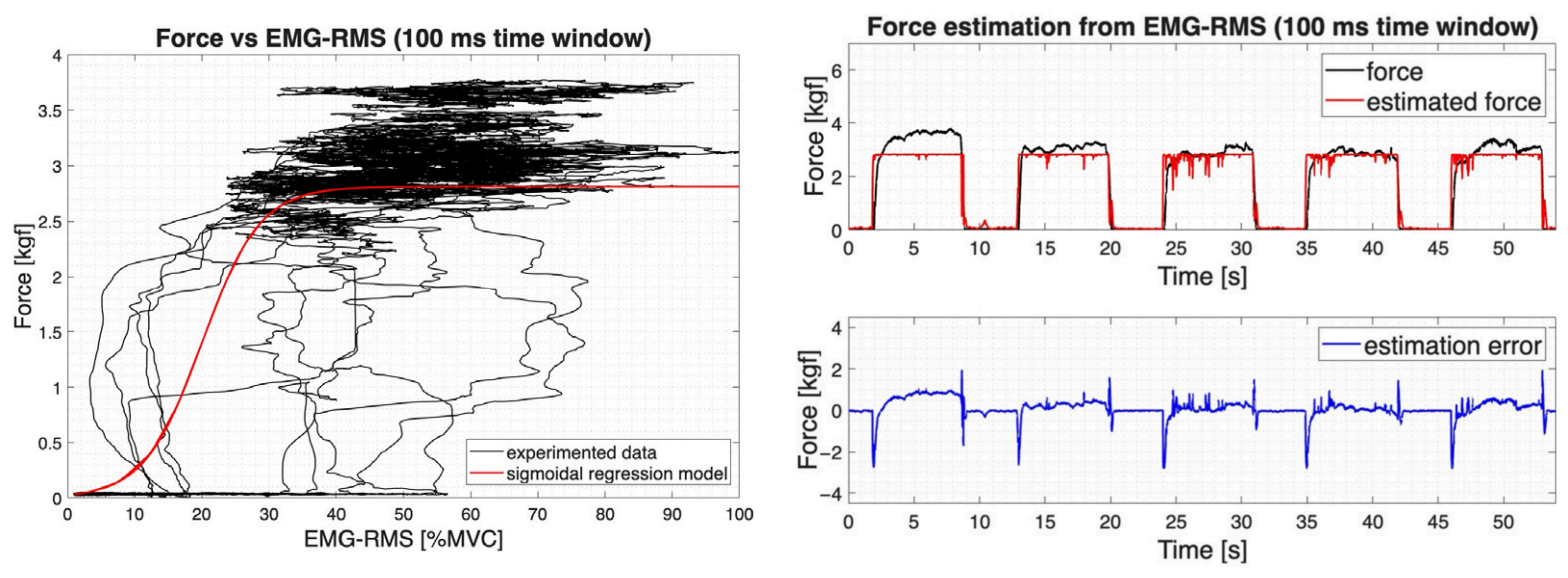
Subject-specific EMG-RMS-based force estimation, using a 100 ms RMS time window for subject #5 during the five held-out test contractions of Test B (rapid increase–decrease isometric contractions). Left: relationship between normalized EMG-RMS and measured force for the five test contractions (black), together with the sigmoidal regression curve fitted using the first 10 contractions (red). Upper right: comparison between the reference force (“force”, black) and the model prediction (“estimated force”, red). Bottom right: estimation error over time, calculated as reference force minus EMG-RMS-based estimated force.

**Figure 12:**
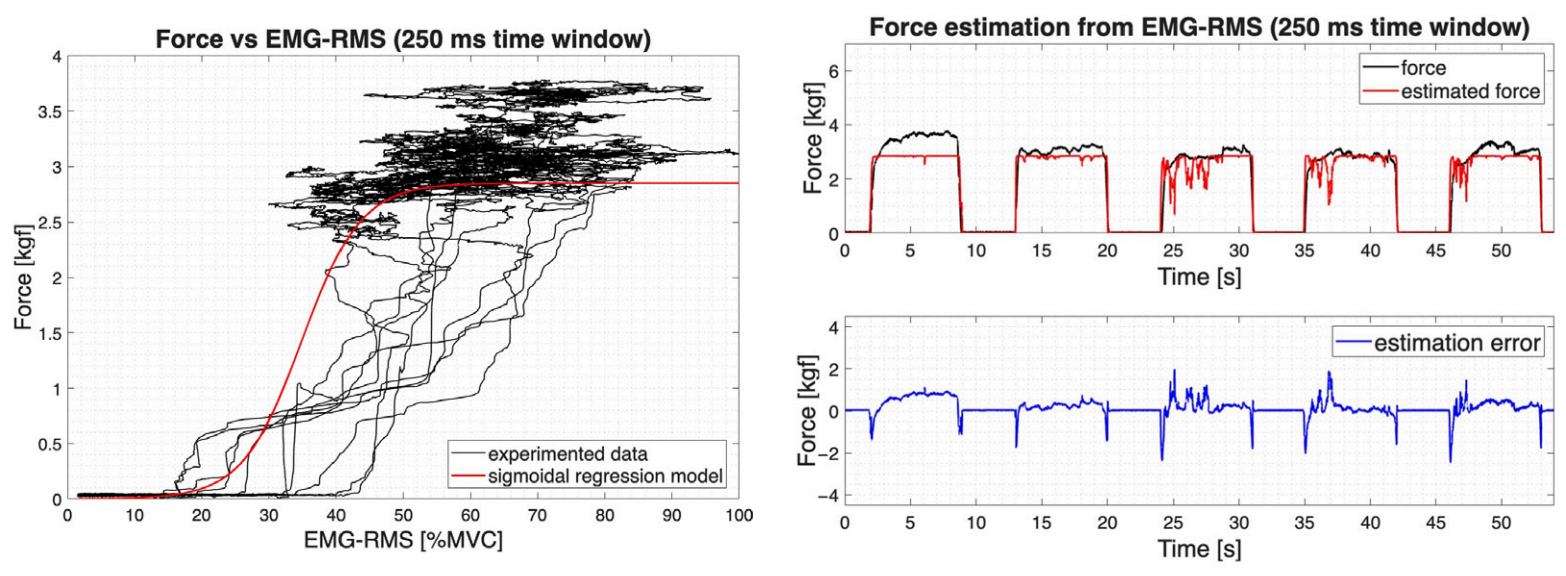
Subject-specific EMG-RMS-based force estimation, using a 250 ms RMS time window for subject #5 during the five held-out test contractions of Test B (rapid increase–decrease isometric contractions). Left: relationship between normalized EMG-RMS and reference force for the five test contractions (black), together with the sigmoidal regression curve fitted using the first 10 contractions (red). Upper right: comparison between the measured force (“force”, black) and the model prediction (“estimated force”, red). Bottom right: estimation error over time, calculated as reference force minus EMG-RMS-based estimated force.

**Figure 13:**
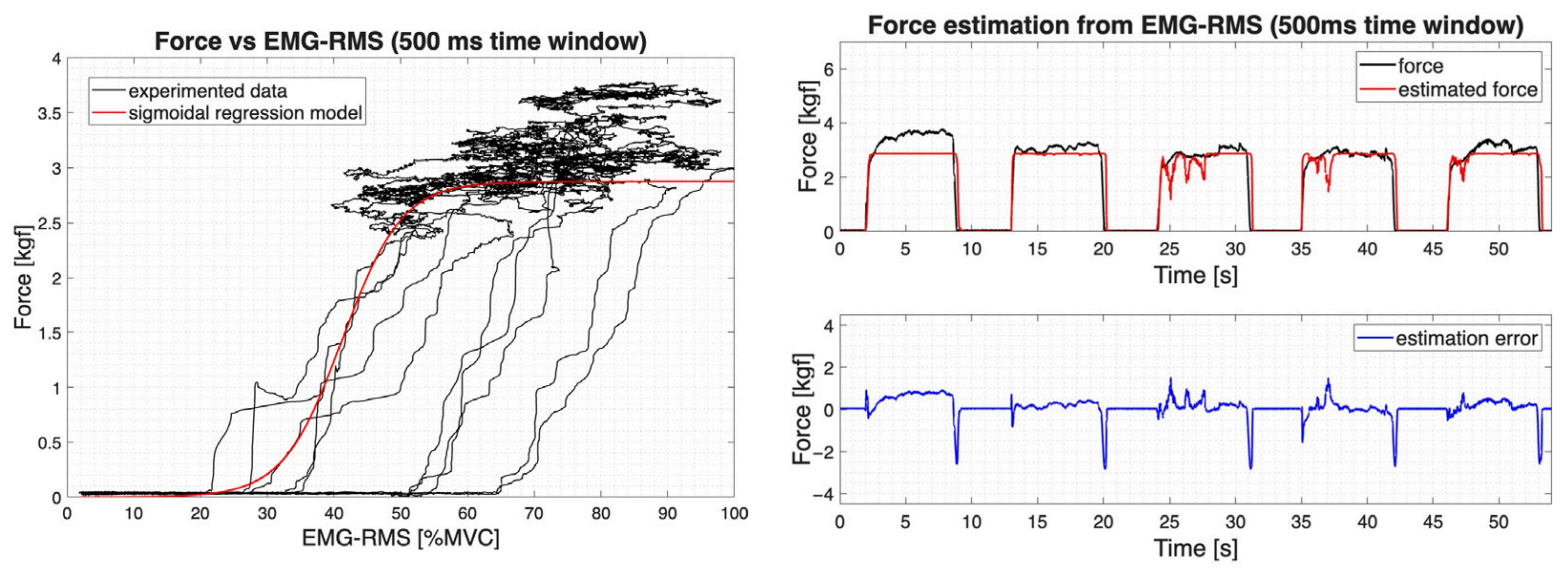
Subject-specific EMG-RMS-based force estimation, using a 500 ms RMS time window for subject #5 during the five held-out test contractions of Test B (rapid increase–decrease isometric contractions). Left: relationship between normalized EMG-RMS and measured force for the five test contractions (black), together with the sigmoidal regression curve fitted using the first 10 contractions (red). Upper right: comparison between the reference force (“force”, black) and the model prediction (“estimated force”, red). Bottom right: estimation error over time, calculated as reference force minus EMG-RMS-based estimated force.

**Figure 14:**
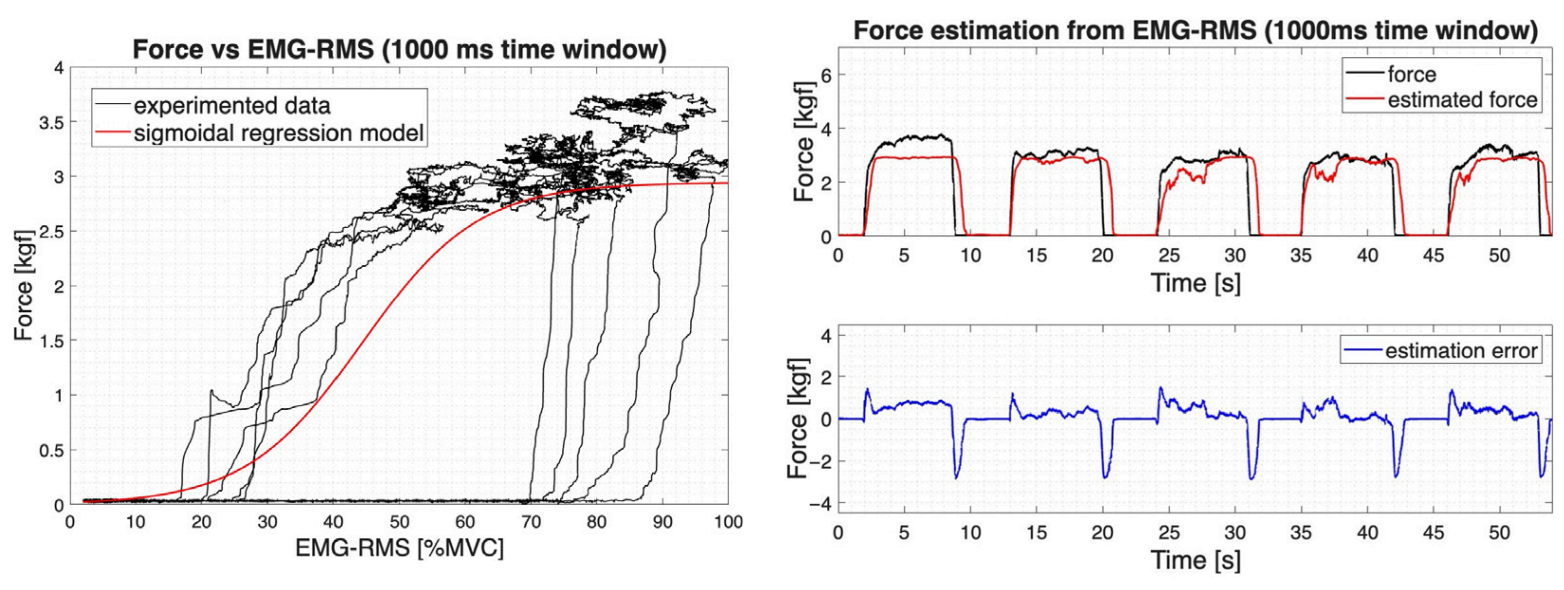
Subject-specific EMG-RMS-based force estimation, using a 1000 ms RMS time window for subject #5 during the five held-out test contractions of Test B (rapid increase–decrease isometric contractions). Left: relationship between normalized EMG-RMS and measured force for the five test contractions (black), together with the sigmoidal regression curve fitted using the first 10 contractions (red). Upper right: comparison between the reference force (“force”, black) and the model prediction (“estimated force”, red). Bottom right: estimation error over time, calculated as reference force minus EMG-RMS-based estimated force.

Figure 11, Figure 12, Figure 13 and Figure 14 illustrate the performance of the subject-specific EMG-RMS regression models for subject #5 during Test B. In each figure, the left panel shows the relationship between the normalized EMG-RMS signal, computed using time windows ranging from 100 ms to 1 s, and the force signal, together with the fitted sigmoidal regression model. The right panels show the corresponding force estimation results: the upper panel compares the force signal with the EMG-RMS-based estimated force, while the bottom panel reports the estimation error over time, computed as the difference between the force signal and the EMG-RMS-based estimated force.

### 3.3. Statistical Analysis Results

Table 4 and Table 5 summarize the statistical comparisons between FMG- and EMG-RMS-based force estimation during gradual (Test A) and rapid (Test B) contractions, respectively. Each table reports mean paired differences with their 95% bootstrap confidence intervals, together with unadjusted Wilcoxon *p*-values and Bonferroni-adjusted *p*-values. Positive mean differences indicate better FMG performance. Statistical significance was assessed after Bonferroni correction across the 12 comparisons within each contraction condition.

During gradual contractions (Test A, Table 4), mean differences favored FMG in all comparisons. However, only three of the 12 comparisons remained statistically significant after correction, all involving MaAE: 100 ms (adjusted *p* = 0.023), 250 ms (adjusted *p* = 0.047), and 1000 ms (adjusted *p* = 0.023). The corresponding mean reductions in MaAE were 1.173 kgf (95% CI [0.777, 1.565]), 0.692 kgf ([0.384, 1.012]), and 0.806 kgf ([0.469, 1.251]), respectively. No differences in R^2^ or RMSE reached statistical significance after correction.

During rapid contractions (Test B, Table 5), mean differences also favored FMG in all comparisons, and eight of the 12 comparisons remained statistically significant after correction. These comprised R^2^ at 100, 500, and 1000 ms; RMSE at 500 and 1000 ms; and MaAE at 100, 500, and 1000 ms. The largest mean differences in R^2^ and RMSE occurred with the 1000 ms window: an increase of 0.149 in R^2^ (95% CI [0.128, 0.172]) and a reduction of 0.340 kgf in RMSE ([0.257, 0.423]). No comparison with the 250 ms EMG-RMS window reached statistical significance after correction.

These results support an FMG advantage in specific comparisons with the investigated EMG-RMS pipelines, with significant differences observed across more metrics and window lengths during rapid contractions. Nonsignificant comparisons should not be interpreted as evidence of equivalence between methods.

### 3.4. Force-Estimation Lags During Gradual and Rapid Muscle Contractions

**Figure 15:**
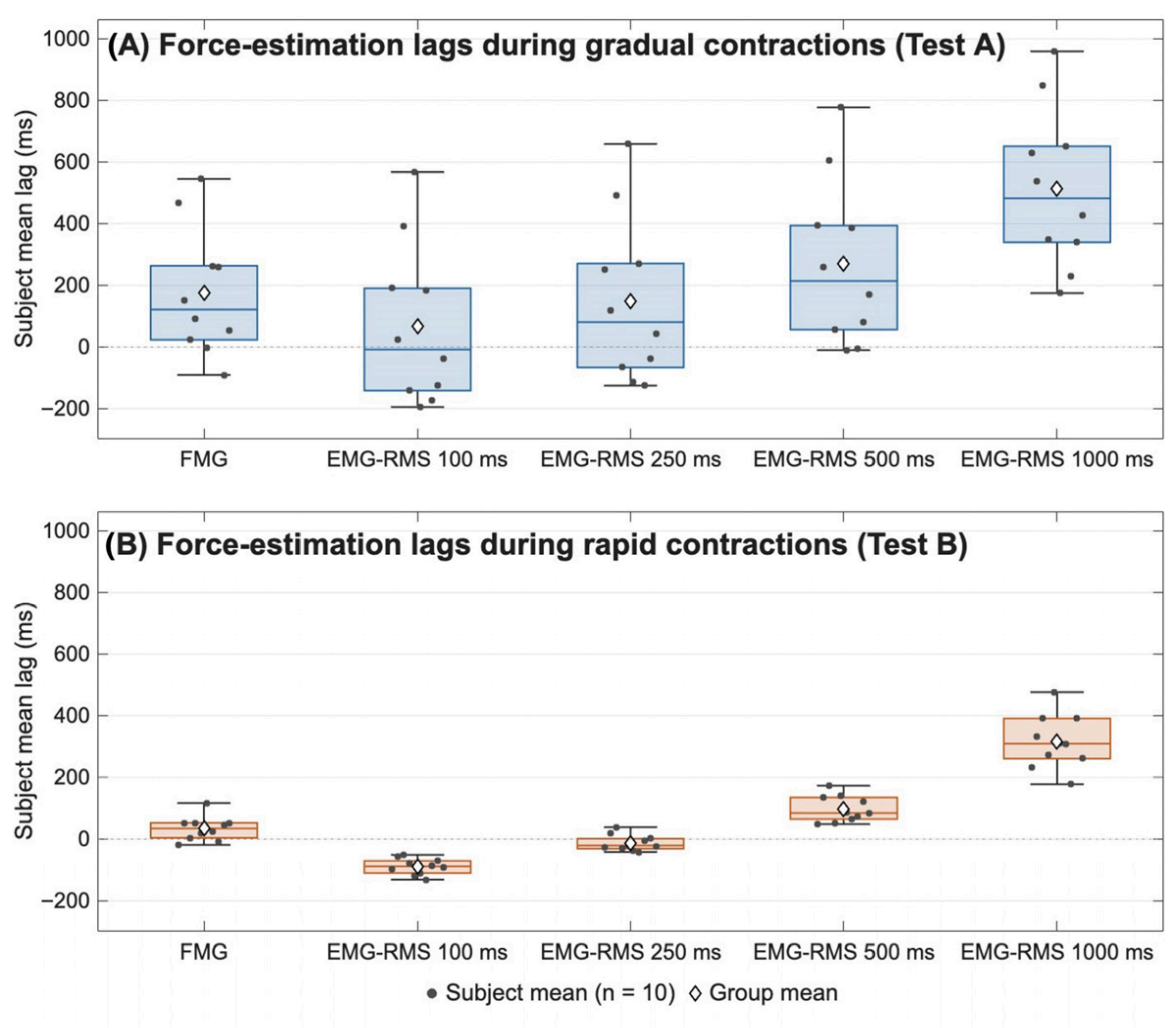
Cross-correlation lags between estimated force and the reference force measured by the load cell during (**A**) gradual contractions (Test A) and (**B**) rapid contractions (Test B). Results are shown for FMG and EMG-RMS force estimators using RMS windows of 100, 250, 500, and 1000 ms. Each grey point represents one subject’s mean lag across the five held-out contractions in the corresponding test dataset (*n* = 10 subjects). Boxes indicate the interquartile range, horizontal lines represent the median, and whiskers extend to the most extreme values within 1.5 times the interquartile range from the quartiles. White diamonds indicate the unweighted mean of the 10-subject means. Positive lags indicate that the estimated force is delayed relative to the reference force signal, whereas negative lags indicate an advance. All lag values are expressed in milliseconds. Subject-specific statistics are reported in Tables S5–S8.

Figure 15 shows the distributions of subject-specific mean lags between the estimated force signals and the reference force measured at the wrist by the load cell during gradual contractions (Test A) and rapid contractions (Test B). The corresponding group statistics are reported in Table 6 as the unweighted mean ± sample standard deviation of the 10 subject-specific means. Positive lags indicate that the estimated force is delayed relative to the reference, whereas negative lags indicate an advance.

During gradual contractions (Figure 15A), the FMG-based estimator exhibited a mean lag of 175.4 ± 206.6 ms. For the EMG-RMS estimators, the group mean lag increased progressively with RMS window duration, from 67.8 ± 259.3 ms for the 100-ms window to 513.9 ± 259.0 ms for the 1000-ms window (Table 6). Substantial between-subject variability was observed, with both positive and negative subject-specific mean lags for FMG and the EMG-RMS estimators using windows of 100–500 ms.

During rapid contractions (Figure 15B), the FMG-based estimator exhibited a smaller group mean lag and lower between-subject variability than in Test A (32.9 ± 39.2 ms). The EMG-RMS estimators showed a transition from negative group mean lags at 100 ms and 250 ms (−90.3 ± 26.1 ms and −13.8 ± 25.9 ms, respectively) to positive lags at 500 ms and 1000 ms (96.9 ± 42.2 ms and 314.8 ± 87.3 ms, respectively). Between-subject standard deviations were lower in Test B than in Test A for every estimator, consistent with the more concentrated distributions shown in Figure 15B.

Subject-specific means, standard deviations, and medians across contractions are reported in Tables S5–S8 of Supplementary Materials.

## 4. Discussion

This study compared FMG and causal EMG-RMS pipelines for estimating the external force exerted at the wrist during gradual and rapid isometric biceps brachii contractions. Earlier FMG–EMG comparisons have addressed control usability, motion detection, and force correlation [32,33,34]. The contribution of the present study is a paired comparison using simultaneous FMG, EMG, and load-cell recordings, four causal RMS window lengths, and separate model-fitting and test contractions. Combining prediction-error metrics with cross-correlation lags characterizes both force agreement and temporal alignment under a controlled posture. Mean prediction performance favored FMG across the tested configurations, while statistical significance and temporal alignment depended on contraction type and RMS window length.

The hold-out design distinguishes the reported prediction performance from in-sample goodness-of-fit. For each participant and contraction type, the first 10 contractions were used for fitting, and the final five were evaluated with fixed model parameters. Signal offsets and MVC normalization references were also fixed without using the held-out contractions. Simultaneous acquisition and a common regression framework allowed comparisons against the same measured force. This design assesses additional contractions of the same participant and type within the investigated recording conditions; it does not establish transfer to new participants, another contraction type, or independent sessions.

The sigmoidal function provided a common empirical model for the nonlinear signal–force relationships. Previous work on EMG–force relationships and motor-unit behavior informed this choice [15,41], but the fitted curves do not establish a physiological mechanism for the FMG–force relationship. Moreover, FMG underwent baseline correction and MVC normalization, whereas EMG additionally underwent RMS-envelope extraction. The comparison consequently reflects the combined sensing, preprocessing, and regression configurations.

After Bonferroni correction across 12 comparisons within each contraction condition, the statistical evidence was more selective than the differences in group means alone suggest. During gradual contractions, FMG had significantly lower MaAE than EMG-RMS at 100, 250, and 1000 ms, while no R^2^ or RMSE comparison remained significant (Table 4). During rapid contractions, significant differences favored FMG for R^2^ at 100, 500, and 1000 ms, RMSE at 500 and 1000 ms, and MaAE at 100, 500, and 1000 ms (Table 5). None of the three comparisons with the 250 ms window was significant during rapid contractions. Thus, the data support an FMG advantage in specific comparisons, with significant differences across more metrics and windows during rapid contractions. Nonsignificant results do not establish equivalence, and these results do not imply that FMG outperforms EMG irrespective of its processing strategy.

The mean paired differences and their confidence intervals quantify the magnitude and uncertainty of these effects. These pointwise bootstrap intervals were not adjusted for multiple comparisons and complement, rather than replace, the corrected Wilcoxon tests. The independent sample remained ten participants; repeated contractions and bootstrap resampling do not increase that number. The participant-specific results in Tables S1–S4 also show variability that limits the interpretation of group averages.

The lag analysis does not support a universal temporal advantage of FMG (Figure 15 and Table 6). During gradual contractions, the FMG group mean lag was 175.4 ± 206.6 ms, while EMG-RMS group means ranged from 67.8 ± 259.3 ms at 100 ms to 513.9 ± 259.0 ms at 1000 ms. During rapid contractions, the FMG lag was 32.9 ± 39.2 ms; EMG-RMS group means were −90.3 ± 26.1, −13.8 ± 25.9, 96.9 ± 42.2, and 314.8 ± 87.3 ms for the four increasing window lengths. Positive values denote delay and negative values denote advance relative to the reference force signal. In rapid contractions, the signed group mean for the 250 ms window was closer to zero than that for FMG, although this is not a comparison of mean absolute participant-level lags. These descriptive results, together with Tables S5–S8, show that temporal alignment depends on both the estimator and the contraction profile.

Longer RMS windows produced smoother envelopes and progressively more positive group mean lags, consistent with a contribution of temporal smoothing to force-tracking differences. Cross-correlation lags combine physiological, sensor, and estimation characteristics and do not measure software execution time. Electromechanical delay concerns the interval between electrical activation and mechanical force development [44] and was not independently measured here. The analysis was performed offline using causal RMS windows; acquisition-to-output latency, including buffering and computation, was not quantified. The 500 and 1000 ms windows characterize the smoothing trade-off, but their inclusion does not establish suitability for responsive control.

All performance metrics quantify agreement with external wrist force, not a direct measurement of biceps brachii force. The simplified lever model in Section 2.1 provides an interpretation of equivalent muscle force under stated geometric and static assumptions, but its scaling was not applied to the reported predictions or errors. The model omits gravitational moments and the contributions of synergistic and antagonist muscles and uses a fixed anatomical parameter for the biceps insertion. More detailed participant-specific anatomy and independent biomechanical validation would be required to assess the accuracy of equivalent muscle-force estimates.

The protocol also limits comparisons between contraction conditions. Because contraction intensity was self-selected, force levels were not standardized across repetitions or participants. Test A always preceded Test B, and fatigue was not separately assessed. The rest intervals allowed recovery but do not demonstrate the absence of fatigue or order effects.

FMG performance also depends on mechanical coupling. The elastic band was adjusted manually, without a predefined contact-force target or quantitative standardization of strap tension. Differences in contact conditions may therefore have influenced the baseline and sensor sensitivity. Baseline subtraction, MVC normalization, and participant-specific regression account for the recorded signals under the calibrated conditions but do not establish robustness to changes in coupling. Repeatability after repositioning and transfer across contact conditions or recording sessions were not assessed. These factors should be tested before assuming that a fitted model remains valid after the sensor is removed and reapplied.

The practical meaning of the errors depends on the intended force range and control requirements. FMG mean participant-level RMSE was 0.422 kgf during gradual contractions and 0.382 kgf during rapid contractions, whereas mean participant-level MaAE was 1.360 and 2.029 kgf, respectively. Thus, low overall RMSE can coexist with larger individual deviations. Self-selected contraction intensities prevent assuming a common prescribed force range, and participant-specific test ranges and range-normalized errors would provide useful additional context.

The cohort comprised only ten healthy participants, and all measurements concerned isometric contractions at approximately 90° elbow flexion. This controlled posture reduced variation in joint kinematics but did not test changing muscle length, joint angle, or dynamic loading. Larger and more heterogeneous cohorts, including relevant clinical populations, are needed to assess generalizability. The use of one regression family also limits the comparison: alternative EMG features, envelope methods, and regression approaches may produce different results. Data-driven force-estimation methods [45,46] warrant comparison using independent evaluation data and the same operational constraints.

FMG offers practical features worth exploring for wearable force-proportional interfaces. The present setup used a thin piezoresistive sensor with simple conditioning circuitry [29], and FMG avoided the RMS-envelope extraction used in the EMG pipelines. These features motivate further development, although comfort, long-term stability, energy consumption, and total computational burden were not compared in this experiment. The relevance to HMI design lies in the measured force-tracking performance and the identified dependence on contraction profile and processing window. Practical deployment in HMIs, rehabilitation devices, or assistive exoskeletons requires validation during dynamic tasks, across sessions and sensor contact conditions, together with acquisition-to-output timing measurements and closed-loop testing in the intended users.

## 5. Conclusions

This study supports FMG as an effective alternative to the investigated EMG-RMS pipelines for estimating external wrist force during isometric biceps brachii contractions. In ten healthy participants, participant-specific FMG models achieved higher mean R^2^ and lower mean RMSE and MaAE across all tested RMS windows during both gradual and rapid contractions. These findings were obtained on held-out contractions, supporting prediction beyond the data used for calibration. Statistical evidence favoring FMG was strongest during rapid contractions, with eight of twelve comparisons remaining significant after Bonferroni correction.

A key strength of FMG is that these results were achieved without RMS-envelope extraction, using baseline correction, MVC normalization, and sigmoidal regression. FMG therefore combines favorable force-estimation performance with simpler preprocessing and an electrode-free sensing arrangement. During rapid contractions, FMG also showed smaller positive mean lags than the 500 and 1000 ms EMG-RMS pipelines. The benefits remain configuration-dependent: the comparisons with the 250 ms EMG-RMS window were not significant during rapid contractions, and a universal timing advantage was not established. Within these boundaries, FMG is a promising candidate for force-proportional HMIs, rehabilitation devices, and assistive exoskeletons. Future validation should address dynamic tasks, repeated sessions, sensor repositioning, and end-to-end performance in closed-loop use.

## Figures and Tables

**Table 1:** Participant characteristics.

Subject	Age [Years]	Gender	BMI [kg/m^2^]	k [Dimensionless]
#1	24	F	19.56	7.61
#2	25	F	23.11	6.57
#3	20	M	29.41	8.66
#4	19	M	23.94	8.95
#5	27	M	26.94	8.21
#6	29	M	30.49	7.76
#7	24	M	21.63	8.06
#8	24	F	19.59	8.95
#9	25	F	22.55	7.61
#10	29	F	20.57	8.36

BMI: body mass index; F: female; M: male; k: dimensionless participant-specific scaling factor used in Equation (2).

**Table 2:** Performance of the EMG-RMS-based force estimation during gradual increase–decrease isometric muscle contractions (Test A). Subject-specific sigmoidal regression models relating the force signal to EMG-RMS signals computed using RMS time windows of 100, 250, 500, and 1000 ms. The table reports the mean ± standard deviation of the coefficient of determination (R^2^), Root Mean Squared Error (RMSE), and Maximum Absolute Error (MaAE) across all ten subjects.

RMS Window	R^2^ (Mean ± SD)	RMSE [kgf] (Mean ± SD)	MaAE [kgf] (Mean ± SD)
100 ms	0.875 ± 0.062	0.548 ± 0.191	2.534 ± 0.721
250 ms	0.892 ± 0.057	0.499 ± 0.169	2.052 ± 0.472
500 ms	0.893 ± 0.049	0.489 ± 0.128	1.844 ± 0.397
1000 ms	0.860 ± 0.058	0.570 ± 0.153	2.166 ± 0.677

**Table 3:** Performance of the EMG-RMS-based force estimation during rapid increase–decrease isometric muscle contractions (Test B). Subject-specific sigmoidal regression models relating the force signal to EMG-RMS signals computed using RMS time windows of 100, 250, 500, and 1000 ms. The table reports the mean ± standard deviation of the coefficient of determination (R^2^), Root Mean Squared Error (RMSE), and Maximum Absolute Error (MaAE) across all ten subjects.

RMS Window	R^2^ (Mean ± SD)	RMSE [kgf] (Mean ± SD)	MaAE [kgf] (Mean ± SD)
100 ms	0.895 ± 0.034	0.497 ± 0.173	3.082 ± 1.277
250 ms	0.918 ± 0.024	0.440 ± 0.147	2.778 ± 1.189
500 ms	0.896 ± 0.022	0.504 ± 0.178	3.065 ± 1.259
1000 ms	0.794 ± 0.030	0.722 ± 0.290	3.178 ± 1.232

**Table 4:** Comparison of FMG- and EMG-RMS-based force estimation during gradual isometric contractions (Test A), using participant-level metrics from five held-out contractions (*n* = 10 subjects). Mean paired differences are FMG−EMG-RMS for R^2^ and EMG-RMS−FMG for RMSE and MaAE; positive values favor FMG. R^2^ differences are dimensionless; error differences are in kgf. The 95% CIs are percentile bootstrap intervals based on 50,000 participant-level resamples, without multiplicity adjustment. Exact, two-sided paired Wilcoxon *p*-values and Bonferroni-adjusted *p*-values for the 12 comparisons are reported; adjusted *p* < 0.05 indicates statistical significance.

Metric	RMS Window (ms)	Mean Difference	95% CI	Wilcoxon *p*-Value	Bonferroni-Adjusted *p*-Value
R^2^	100	0.047	[0.005, 0.092]	0.105	1
	250	0.030	[−0.005, 0.073]	0.186	1
	500	0.029	[−0.004, 0.066]	0.232	1
	1000	0.062	[0.026, 0.102]	0.010	0.117
RMSE	100	0.126	[0.023, 0.242]	0.064	0.773
	250	0.077	[−0.018, 0.195]	0.160	1
	500	0.067	[−0.022, 0.170]	0.275	1
	1000	0.147	[0.061, 0.253]	0.010	0.117
MaAE	100	1.173	[0.777, 1.565]	0.002	0.023
	250	0.692	[0.384, 1.012]	0.004	0.047
	500	0.484	[0.199, 0.781]	0.014	0.164
	1000	0.806	[0.469, 1.251]	0.002	0.023

**Table 5:** Comparison of FMG and EMG-RMS-based force estimation during rapid isometric contractions (Test B), using participant-level metrics from five held-out contractions (*n* = 10 subjects). Mean paired differences are FMG − EMG-RMS for R^2^ and EMG-RMS − FMG for RMSE and MaAE; positive values favor FMG. R^2^ differences are dimensionless; error differences are in kgf. The 95% CIs are percentile bootstrap intervals based on 50,000 participant-level resamples, without multiplicity adjustment. Exact, two-sided paired Wilcoxon *p*-values and Bonferroni-adjusted *p*-values for the 12 comparisons are reported; adjusted *p* < 0.05 indicates statistical significance.

Metric	RMS Window (ms)	Mean Difference	95% CI	Wilcoxon *p*-Value	Bonferroni-Adjusted *p*-Value
R^2^	100	0.049	[0.027, 0.071]	0.004	0.047
	250	0.025	[0.010, 0.040]	0.064	0.773
	500	0.047	[0.032, 0.063]	0.002	0.023
	1000	0.149	[0.128, 0.172]	0.002	0.023
RMSE	100	0.116	[0.066, 0.165]	0.006	0.070
	250	0.058	[0.020, 0.100]	0.064	0.773
	500	0.122	[0.084, 0.162]	0.002	0.023
	1000	0.340	[0.257, 0.423]	0.002	0.023
MaAE	100	1.053	[0.647, 1.464]	0.004	0.047
	250	0.749	[0.296, 1.228]	0.020	0.234
	500	1.035	[0.602, 1.502]	0.004	0.047
	1000	1.149	[0.748, 1.568]	0.004	0.047

**Table 6:** Group-level cross-correlation lags between each force estimator and the load-cell reference during gradual contractions (Test A) and rapid contractions (Test B). Values are expressed as the unweighted mean ± sample standard deviation of the 10 subject-specific mean lags. The standard deviation represents between-subject variability. All values are expressed in milliseconds.

Force Estimator	Test A: Mean ± SD (ms)	Test B: Mean ± SD (ms)
FMG	175.4 ± 206.6	32.9 ± 39.2
EMG-RMS 100 ms	67.8 ± 259.3	−90.3 ± 26.1
EMG-RMS 250 ms	148.4 ± 267.0	−13.8 ± 25.9
EMG-RMS 500 ms	270.8 ± 267.4	96.9 ± 42.2
EMG-RMS 1000 ms	513.9 ± 259.0	314.8 ± 87.3

## Data Availability

The data supporting this study are available upon request to the corresponding author.

## Supplementary Materials

The following supporting information can be downloaded at: https://www.mdpi.com/article/10.3390/s26206543/s1, Table S1. Subject-specific FMG-based force estimation performance during gradual increase–decrease isometric contractions (Test A). For each participant, the coefficient of determination (R^2^), root mean squared error (RMSE), and maximum absolute error (MaAE) were calculated on the final five held-out test contractions. The sigmoidal regression model was fitted using the first 10 contractions of the same participant and condition and applied to the test data without refitting or parameter adjustment. R^2^ is dimensionless; RMSE and MaAE are expressed in kgf and refer to the external force measured at the wrist by the load cell. No conversion to equivalent biceps brachii force was applied. Table S2: Subject-specific EMG-RMS-based force estimation performance during gradual increase–decrease isometric contractions (Test A), using RMS windows of 100, 250, 500, and 1000 ms. For each participant and RMS window, the coefficient of determination (R^2^), root mean squared error (RMSE), and maximum absolute error (MaAE) were calculated on the final five held-out test contractions. Each sigmoidal regression model was fitted using the first 10 contractions of the same participant and condition and applied to the test data without refitting or parameter adjustment. R^2^ is dimensionless; RMSE and MaAE are expressed in kgf and refer to the external force measured at the wrist by the load cell. No conversion to equivalent biceps brachii force was applied. Table S3: Subject-specific FMG-based force estimation performance during rapid increase–decrease isometric contractions (Test B). For each participant, the coefficient of determination (R^2^), root mean squared error (RMSE), and maximum absolute error (MaAE) were calculated on the final five held-out test contractions. The sigmoidal regression model was fitted using the first 10 contractions of the same participant and condition and applied to the test data without refitting or parameter adjustment. R^2^ is dimensionless; RMSE and MaAE are expressed in kgf and refer to the external force measured at the wrist by the load cell. No conversion to equivalent biceps brachii force was applied. Table S4: Subject-specific EMG-RMS-based force estimation performance during rapid increase–decrease isometric contractions (Test B), using RMS windows of 100, 250, 500, and 1000 ms. For each participant and RMS window, the coefficient of determination (R^2^), root mean squared error (RMSE), and maximum absolute error (MaAE) were calculated on the final five held-out test contractions. Each sigmoidal regression model was fitted using the first 10 contractions of the same participant and condition and applied to the test data without refitting or parameter adjustment. R^2^ is dimensionless; RMSE and MaAE are expressed in kgf and refer to the external force measured at the wrist by the load cell. No conversion to equivalent biceps brachii force was applied. Table S5: Subject-specific cross-correlation lags between the FMG-based force estimate and the reference force measured at the wrist by the load cell during test A (gradual contractions). Mean, standard deviation, and median summarize the lags obtained across the analyzed contractions in each subject’s test dataset (five muscle contractions). All values are expressed in milliseconds. Positive lags indicate that the FMG-based force estimate is delayed relative to the load-cell reference, whereas negative lags indicate an advance. Table S6: Subject-specific cross-correlation lags between the EMG-RMS-based force estimates and the reference force measured at the wrist by the load cell during test A (gradual contractions). Results are reported for RMS time windows of 100, 250, 500, and 1000 ms. For each subject and RMS window, the mean, standard deviation, and median summarize the lags obtained across the analyzed contractions in the test dataset (five muscle contractions). All lag values are expressed in milliseconds. Positive lags indicate that the EMG-RMS-based force estimate is delayed relative to the load-cell reference, whereas negative lags indicate an advance. Table S7: Subject-specific cross-correlation lags between the FMG-based force estimate and the reference force measured at the wrist by the load cell during test B (rapid contractions). Mean, standard deviation, and median summarize the lags obtained across the analyzed contractions in each subject’s test dataset (five muscle contractions). All values are expressed in milliseconds. Positive lags indicate that the FMG-based force estimate is delayed relative to the load-cell reference, whereas negative lags indicate an advance. Table S8: Subject-specific cross-correlation lags between the EMG-RMS-based force estimates and the reference force measured at the wrist by the load cell during test B (rapid contractions). Results are reported for RMS time windows of 100, 250, 500, and 1000 ms. For each subject and RMS window, the mean, standard deviation, and median summarize the lags obtained across the analyzed contractions in the test dataset (five muscle contractions). All lag values are expressed in milliseconds. Positive lags indicate that the EMG-RMS-based force estimate is delayed relative to the load-cell reference, whereas negative lags indicate an advance.

## Abbreviations

The following abbreviations are used in this manuscript:

BMI	Body Mass Index
CI	Confidence Interval
DAQ	Data Acquisition
EMG	Electromyography
FMG	Forcemyography
FSR	Force Sensitive Resistor
HMI	Human–Machine Interface
MaAE	Maximum Absolute Error
MVC	Maximum Voluntary Contraction
R^2^	Coefficient of Determination
RMS	Root Mean Square
RMSE	Root Mean Squared Error
sEMG	Surface Electromyography
