# Supplementary material for: A Performance Comparison of Forcemyography and Electromyography in Muscle Force Estimation for Human–Machine Interfaces
Source: Sensors (Basel). 2026 Oct 15;26(20):6543. doi: 10.3390/s26206543 (PMC13709319; doi:10.3390/s26206543)
Supplement: Supplementary file 1 [file sensors-26-06543-s001.zip › sensors-4499335-supplementary.pdf]

A Performance Comparison of Forcemyography and Electromyography in Muscle Force Estimation for Human-Machine Interfaces

Supplementary Material

Subject-specific tables supporting the comparison of FMG and EMG-RMS for force estimation.

Table S1. Subject-specific FMG-based force estimation performance during gradual increase–decrease isometric contractions (Test A). For each participant, the coefficient of determination (R^2), root mean squared error (RMSE), and maximum absolute error (MaAE) were calculated on the final five held-out test contractions. The sigmoidal regression model was fitted using the first 10 contractions of the same participant and condition and applied to the test data without refitting or parameter adjustment. R^2 is dimensionless; RMSE and MaAE are expressed in kgf and refer to the external force measured at the wrist by the load cell. No conversion to equivalent biceps brachii force was applied.

Subject	R^2	RMSE [kgf]	MaAE [kgf]
#1	0.930	0.280	1.044
#2	0.887	0.617	1.773
#3	0.970	0.305	1.104
#4	0.975	0.188	0.826
#5	0.921	0.347	1.056
#6	0.948	0.612	1.510
#7	0.842	0.502	1.638
#8	0.853	0.631	1.901
#9	0.930	0.350	1.402
#10	0.965	0.391	1.349

Table S2. Subject-specific EMG-RMS-based force estimation performance during gradual increase–decrease isometric contractions (Test A), using RMS windows of 100, 250, 500, and 1000 ms. For each participant and RMS window, the coefficient of determination (R^2), root mean squared error (RMSE), and maximum absolute error (MaAE) were calculated on the final five held-out test contractions. Each sigmoidal regression model was fitted using the first 10 contractions of the same participant and condition and applied to the test data without refitting or parameter adjustment. R^2 is dimensionless; RMSE and MaAE are expressed in kgf and refer to the external force measured at the wrist by the load cell. No conversion to equivalent biceps brachii force was applied.

Subject	RMS time window	R^2	RMSE [kgf]	MaAE [kgf]
#1	100 ms	0.914	0.311	1.647
	250 ms	0.928	0.284	1.565
	500 ms	0.891	0.350	1.701
	1000 ms	0.846	0.416	1.789
#2	100 ms	0.921	0.517	3.489
	250 ms	0.939	0.452	2.583
	500 ms	0.941	0.445	1.652
	1000 ms	0.908	0.556	2.180
#3	100 ms	0.785	0.824	3.180
	250 ms	0.793	0.808	2.756
	500 ms	0.835	0.721	2.131
	1000 ms	0.782	0.830	2.180
#4	100 ms	0.838	0.480	2.445
	250 ms	0.866	0.438	2.183
	500 ms	0.860	0.445	2.041
	1000 ms	0.844	0.471	1.965
#5	100 ms	0.869	0.446	1.844
	250 ms	0.878	0.430	1.632
	500 ms	0.861	0.459	1.481
	1000 ms	0.818	0.525	1.922
#6	100 ms	0.918	0.769	3.499
	250 ms	0.935	0.683	2.377
	500 ms	0.943	0.643	2.660
	1000 ms	0.913	0.793	4.019
#7	100 ms	0.899	0.400	1.984
	250 ms	0.837	0.509	1.945
	500 ms	0.830	0.520	1.755
	1000 ms	0.784	0.586	1.961
#8	100 ms	0.757	0.810	2.773
	250 ms	0.842	0.655	2.158
	500 ms	0.870	0.593	2.144
	1000 ms	0.838	0.663	2.176
#9	100 ms	0.929	0.352	1.687
	250 ms	0.952	0.291	1.236
	500 ms	0.934	0.341	1.350
	1000 ms	0.927	0.359	1.870
#10	100 ms	0.924	0.574	2.788
	250 ms	0.954	0.446	2.084
	500 ms	0.967	0.376	1.528
	1000 ms	0.943	0.497	1.602

Table S3. Subject-specific FMG-based force estimation performance during rapid increase–decrease isometric contractions (Test B). For each participant, the coefficient of determination (R^2), root mean squared error (RMSE), and maximum absolute error (MaAE) were calculated on the final five held-out test contractions. The sigmoidal regression model was fitted using the first 10 contractions of the same participant and condition and applied to the test data without refitting or parameter adjustment. R^2 is dimensionless; RMSE and MaAE are expressed in kgf and refer to the external force measured at the wrist by the load cell. No conversion to equivalent biceps brachii force was applied.

**Subject**	**R^2**	**RMSE [kgf]**	**MaAE [kgf]**
**#1**	0.929	0.310	1.189
**#2**	0.930	0.676	3.936
**#3**	0.940	0.439	1.618
**#4**	0.919	0.375	2.443
**#5**	0.971	0.249	0.675
**#6**	0.947	0.592	3.640
**#7**	0.958	0.106	0.567
**#8**	0.963	0.283	1.828
**#9**	0.938	0.297	1.592
**#10**	0.936	0.492	2.806

Table S4. Subject-specific EMG-RMS-based force estimation performance during rapid increase–decrease isometric contractions (Test B), using RMS windows of 100, 250, 500, and 1000 ms. For each participant and RMS window, the coefficient of determination (R^2), root mean squared error (RMSE), and maximum absolute error (MaAE) were calculated on the final five held-out test contractions. Each sigmoidal regression model was fitted using the first 10 contractions of the same participant and condition and applied to the test data without refitting or parameter adjustment. R^2 is dimensionless; RMSE and MaAE are expressed in kgf and refer to the external force measured at the wrist by the load cell. No conversion to equivalent biceps brachii force was applied.

Subject	RMS time window	R^2	RMSE [kgf]	MaAE [kgf]
#1	100 ms	0.892	0.382	1.940
	250 ms	0.908	0.352	1.737
	500 ms	0.899	0.369	1.966
	1000 ms	0.835	0.471	2.101
#2	100 ms	0.933	0.659	4.797
	250 ms	0.932	0.666	3.637
	500 ms	0.908	0.773	4.604
	1000 ms	0.776	1.210	5.248
#3	100 ms	0.897	0.578	3.634
	250 ms	0.915	0.523	3.571
	500 ms	0.881	0.621	3.834
	1000 ms	0.770	0.864	3.899
#4	100 ms	0.831	0.542	2.247
	250 ms	0.863	0.488	2.329
	500 ms	0.852	0.508	2.442
	1000 ms	0.770	0.632	2.406
#5	100 ms	0.891	0.480	2.788
	250 ms	0.913	0.428	2.431
	500 ms	0.884	0.496	2.834
	1000 ms	0.757	0.718	2.882
#6	100 ms	0.901	0.811	5.170
	250 ms	0.947	0.593	5.257
	500 ms	0.919	0.735	5.338
	1000 ms	0.815	1.110	4.761
#7	100 ms	0.843	0.205	1.117
	250 ms	0.906	0.159	1.110
	500 ms	0.880	0.179	1.052
	1000 ms	0.758	0.255	1.164
#8	100 ms	0.904	0.459	3.054
	250 ms	0.919	0.420	2.835
	500 ms	0.904	0.459	3.075
	1000 ms	0.824	0.621	3.166
#9	100 ms	0.931	0.314	2.287
	250 ms	0.940	0.294	1.810
	500 ms	0.909	0.360	2.407
	1000 ms	0.808	0.525	2.610
#10	100 ms	0.921	0.545	3.788
	250 ms	0.940	0.473	3.065
	500 ms	0.924	0.533	3.096
	1000 ms	0.825	0.811	3.542

Table S5. Subject-specific cross-correlation lags between the FMG-based force estimate and the reference force measured at the wrist by the load cell during test A (gradual contractions). Mean, standard deviation, and median summarize the lags obtained across the analyzed contractions in each subject's test dataset (five muscle contractions). All values are expressed in milliseconds. Positive lags indicate that the FMG-based force estimate is delayed relative to the load-cell reference, whereas negative lags indicate an advance.

Subject	Mean (ms)	Standard Deviation (ms)	Median (ms)
**#1**	465.6	50.7	456.0
**#2**	151.0	53.7	136.0
**#3**	22.8	45.8	17.0
**#4**	90.5	38.6	84.5
**#5**	544.6	107.5	590.0
**#6**	−4.2	22.1	7.0
**#7**	262.4	127.4	309.0
**#8**	258.0	222.1	267.0
**#9**	−91.6	42.3	−80.0
**#10**	54.4	126.1	7.0

Table S6. Subject-specific cross-correlation lags between the EMG-RMS-based force estimates and the reference force measured at the wrist by the load cell during test A (gradual contractions). Results are reported for RMS time windows of 100, 250, 500, and 1000 ms. For each subject and RMS window, the mean, standard deviation, and median summarize the lags obtained across the analyzed contractions in the test dataset (five muscle contractions). All lag values are expressed in milliseconds. Positive lags indicate that the EMG-RMS-based force estimate is delayed relative to the load-cell reference, whereas negative lags indicate an advance.

Subject	RMS time window	Mean (ms)	Standard Deviation (ms)	Median (ms)
#1	100 ms	190.0	126.2	190.0
	250 ms	250.0	121.6	250.0
	500 ms	393.0	117.1	390.0
	1000 ms	629.0	157.7	589.0
#2	100 ms	22.2	24.7	14.0
	250 ms	118.2	18.3	119.0
	500 ms	257.6	15.9	260.0
	1000 ms	536.2	29.7	531.0
#3	100 ms	391.6	132.3	384.0
	250 ms	492.2	127.5	429.0
	500 ms	604.8	110.6	553.0
	1000 ms	847.4	92.9	827.0
#4	100 ms	−141.7	64.1	−131.5
	250 ms	−66.7	85.1	−67.0
	500 ms	55.7	105.6	42.5
	1000 ms	349.0	168.8	353.5
#5	100 ms	567.0	247.8	516.0
	250 ms	658.0	226.6	616.0
	500 ms	776.8	164.4	776.0
	1000 ms	958.8	92.1	1000.0
#6	100 ms	−195.6	73.8	−214.0
	250 ms	−115.2	67.2	−141.0
	500 ms	−10.8	63.3	−36.0
	1000 ms	174.0	74.2	175.0
#7	100 ms	183.8	165.9	199.0
	250 ms	270.2	129.7	289.0
	500 ms	385.0	112.2	405.0
	1000 ms	650.2	127.7	670.0
#8	100 ms	−173.2	86.2	−203.0
	250 ms	−125.6	87.3	−158.0
	500 ms	−5.0	91.4	−31.0
	1000 ms	228.8	97.3	210.0
#9	100 ms	−125.8	26.7	−133.0
	250 ms	−39.8	30.0	−47.0
	500 ms	80.8	31.4	78.0
	1000 ms	338.8	46.4	340.0
#10	100 ms	−40.0	49.4	−18.0
	250 ms	42.6	64.0	78.0
	500 ms	169.6	71.0	202.0
	1000 ms	426.4	77.7	464.0

Table S7. Subject-specific cross-correlation lags between the FMG-based force estimate and the reference force measured at the wrist by the load cell during test B (rapid contractions). Mean, standard deviation, and median summarize the lags obtained across the analyzed contractions in each subject's test dataset (five muscle contractions). All values are expressed in milliseconds. Positive lags indicate that the FMG-based force estimate is delayed relative to the load-cell reference, whereas negative lags indicate an advance.

**Subject**	**Mean (ms)**	**Standard Deviation (ms)**	**Median (ms)**
**#1**	−20.0	15.9	−17.0
**#2**	51.6	15.8	49.0
**#3**	3.2	11.2	2.0
**#4**	51.6	18.6	49.0
**#5**	17.0	8.5	20.0
**#6**	116.2	18.1	115.0
**#7**	22.7	13.1	27.0
**#8**	−8.4	22.7	−18.0
**#9**	45.0	20.1	36.0
**#10**	50.6	28.3	54.0

Table S8. Subject-specific cross-correlation lags between the EMG-RMS-based force estimates and the reference force measured at the wrist by the load cell during test B (rapid contractions). Results are reported for RMS time windows of 100, 250, 500, and 1000 ms. For each subject and RMS window, the mean, standard deviation, and median summarize the lags obtained across the analyzed contractions in the test dataset (five muscle contractions). All lag values are expressed in milliseconds. Positive lags indicate that the EMG-RMS-based force estimate is delayed relative to the load-cell reference, whereas negative lags indicate an advance.

Subject	RMS time window	Mean (ms)	Standard Deviation (ms)	Median (ms)
#1	100 ms	−99.2	19.7	−93.0
	250 ms	−28.4	27.7	−17.0
	500 ms	47.8	64.0	59.0
	1000 ms	231.0	101.9	254.0
#2	100 ms	−58.6	11.5	−54.0
	250 ms	17.6	23.7	23.0
	500 ms	134.0	38.4	142.0
	1000 ms	330.8	69.3	346.0
#3	100 ms	−52.0	21.5	−52.0
	250 ms	37.4	27.0	47.0
	500 ms	172.4	43.2	161.0
	1000 ms	392.4	88.6	338.0
#4	100 ms	−78.6	13.3	−83.0
	250 ms	−31.6	16.8	−41.0
	500 ms	50.6	24.2	46.0
	1000 ms	273.4	78.0	257.0
#5	100 ms	−121.0	11.6	−119.0
	250 ms	−19.0	10.5	−23.0
	500 ms	139.0	40.6	143.0
	1000 ms	475.4	80.8	440.0
#6	100 ms	−110.8	19.2	−108.0
	250 ms	−40.0	19.6	−41.0
	500 ms	85.8	28.7	81.0
	1000 ms	310.4	43.8	327.0
#7	100 ms	−132.0	27.5	−131.0
	250 ms	−43.0	30.4	−58.0
	500 ms	64.3	43.1	53.0
	1000 ms	306.3	78.5	333.0
#8	100 ms	−86.6	18.8	−83.0
	250 ms	−7.2	34.7	−10.0
	500 ms	72.4	65.2	35.0
	1000 ms	177.0	141.8	125.0
#9	100 ms	−72.0	22.3	−68.0
	250 ms	0.7	26.6	15.0
	500 ms	121.3	57.5	119.0
	1000 ms	390.7	63.5	354.0
#10	100 ms	−92.6	5.9	−92.0
	250 ms	−24.0	9.3	−26.0
	500 ms	81.6	35.5	69.0
	1000 ms	260.2	47.7	261.0
